# C5a induces A549 cell proliferation of non-small cell lung cancer via GDF15 gene activation mediated by GCN5-dependent KLF5 acetylation

**DOI:** 10.1038/s41388-018-0298-9

**Published:** 2018-05-18

**Authors:** Chenhui Zhao, Yongting Li, Wen Qiu, Fengxia He, Weiming Zhang, Dan Zhao, Zhiwei Zhang, Erbao Zhang, Pei Ma, Yiqian Liu, Ling Ma, Fengming Yang, Yingwei Wang, Yongqian Shu

**Affiliations:** 1 Department of Oncology, The First Affiliated Hospital of Nanjing Medical University, Nanjing, China; 2 Department of Immunology, Nanjing Medical University, Nanjing, China; 3 Department of Pathology, The Second Affiliated Hospital of Nanjing Medical University, Nanjing, China; 4 Department of Pathology, The First Affiliated Hospital of Nanjing Medical University, Nanjing, China; 5 Department of Biochemistry and Molecular Biology, Nanjing Medical University, Nanjing, China; 6 Jiangsu Key Lab of Cancer Biomarkers, Prevention and Treatment, Collaborative Innovation Center of Cancer Medicine, Nanjing Medical University, Nanjing, China

## Abstract

Non-small cell lung cancer (NSCLC) is the most common type of lung cancer, and multiple evidence has confirmed that C5a production is elevated in NSCLC microenvironment. Although NSCLC cell proliferation induced by C5a has been reported, the involved mechanism has not been elucidated. In this study, we examined the proliferation-related genes (i.e., KLF5, GCN5, and GDF15) and C5a receptor (C5aR) expression in tumor tissues as well as C5a concentration in plasma of NSCLC patients, and then determined the roles of KLF5, GCN5, and GDF15 in C5a-triggered NSCLC cell proliferation and the related mechanism both in vitro and in vivo. Our results found that the expression of KLF5, GCN5, GDF15, C5aR, and C5a was significantly upregulated in NSCLC patients. Mechanistic exploration in vitro revealed that C5a could facilitate A549 cell proliferation through increasing KLF5, GCN5, and GDF15 expression. Besides, KLF5 and GCN5 could form a complex, binding to GDF15 promoter in a KLF5-dependent manner and leading to GDF15 gene transcription. More importantly, GCN5-mediated KLF5 acetylation contributing to GDF15 gene transcription and cell proliferation upon C5a stimulation, the region (−103 to +58 nt) of GDF15 promoter which KLF5 could bind to, and two new KLF5 lysine sites (K335 and K391) acetylated by GCN5 were identified for the first time. Furthermore, our experiment in vivo demonstrated that the growth of xenograft tumors in BALB/c nude mice was greatly suppressed by the silence of KLF5, GCN5, or GDF15. Collectively, these findings disclose that C5a-driven KLF5–GCN5–GDF15 axis had a critical role in NSCLC proliferation and might serve as targets for NSCLC therapy.

## Introduction

Complement participates in the processes of inflammatory diseases and malignant tumors [1–3]. Recent studies have revealed that C5a is associated with tumor growth [4, 5], and C5a in tumor microenvironment not only acts as a leukocyte chemoattractant [4, 5], but also promotes tumor cell proliferation [6–8]. Non-small cell lung cancer (NSCLC) is the most common type of lung cancer [9]. Although many researchers have uncovered that inflammatory cytokines or mediators, e.g., C5a are involved in NSCLC carcinogenesis and proliferation [10–12], the mechanism of C5a governing NSCLC cell proliferation remains largely unclear.

It is well known that cell proliferation is associated with the upregulation of transcription factors, transcriptional co-activators and pro-proliferation molecules in response to extracellular stimuli [13, 14]. Reportedly, kruppel-like factor 5 (KLF5), as a transcription factor, can boost breast cancer cell proliferation [15], and activate sox4 or HIF-1α transcription via binding to GC-rich DNA sequences, resulting in lung cancer proliferation [16]. Moreover, as a transcriptional co-activator, general control non-depressible (GCN5) potentiates NSCLC growth by increasing E2F1 and cyclin D1 expression [17] and accelerates glioma development as well [18]. Besides, growth differentiation factor 15 (GDF15) also tends to be an oncoprotein contributing to cancer cell proliferation [19–21]. Given that the pro-proliferation function of KLF5, GCN5, and GDF15 has been confirmed, and the earlier stage of our study also found that KLF5, GCN5, GDF15, C5a and C5aR expression increased in NSCLC patients, how C5a triggers NSCLC cell proliferation and the expression of KLF5, GCN5, or GDF15, and what impact of KLF5, GCN5, or GDF15 on NSCLC cell proliferation in response to C5a and the corresponding mechanism need to be clarified.

Acetylated transcription factors have a pivotal role in the development of cancer or other diseases [22–25]. GCN5, which has acetyltransferase activity, can acetylate transcription factors such as E2F1 [17] or c-Myc and regulate target gene expression in hepatocellular carcinoma and colon cancer [26, 27]. However, the role of KLF5 acetylation mediated by GCN5 in regulating KLF5 function and C5a-induced NSCLC cell proliferation are still unexplored.

In this study, we not only examined the levels of KLF5, GCN5, GDF15, C5aR, or C5a in NSCLC samples, but also evaluated the roles of KLF5, GCN5 and GDF15 in C5a-triggered cell proliferation in vitro and xenograft tumor growth in vivo. Additionally, we also assessed the relationship between these genes and mechanism of them in promoting NSCLC cell proliferation exposed to C5a.

## Results

### Examination of proliferation-related genes, C5a and analysis of correlation between KLF5, GCN5, GDF15, or C5aR expression, and clinic-pathological data in NSCLC patients

**Fig. 1:**
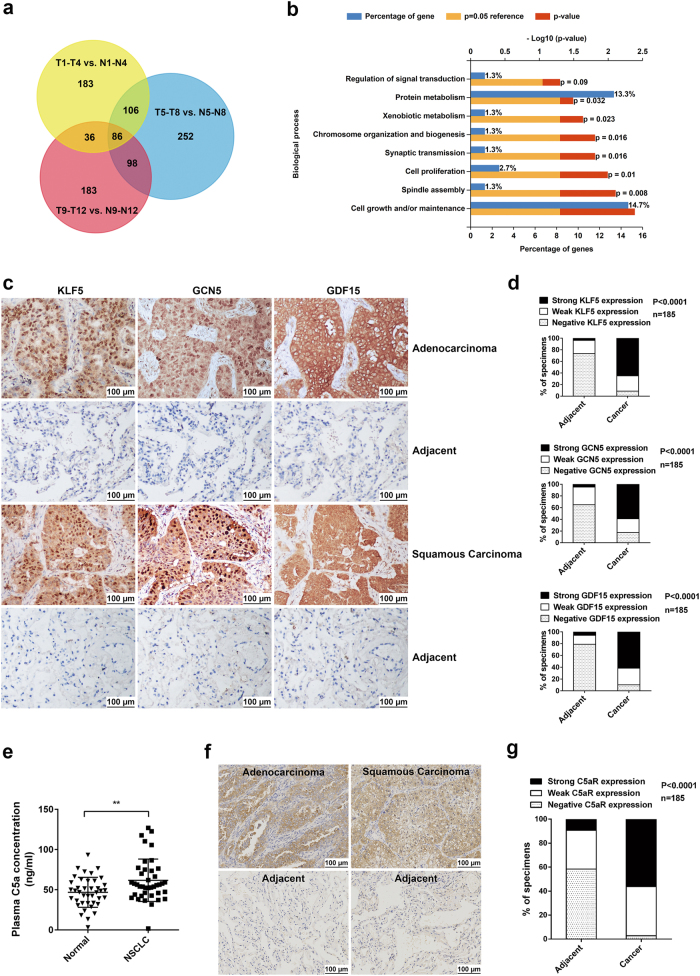
Transcriptome sequencing of NSCLC tissues, the expression of KLF5, GCN5, GDF15, C5aR in tumor tissues and the content of C5a in plasma of NSCLC patients. **a** Venn diagram showed that a total of 944 genes were upregulated (≥2-fold) by RNA-seq of 12 pairs of NSCLC tissues and 86 genes were in the overlap of all three sequencing results. **b** Gene ontology (GO) analysis for the 86 genes displayed that represented biological processes included cell proliferation, growth and maintenance. **c**, **d** Representative images of IHC staining for KLF5, GCN5, and GDF15 in paraffin-embedded sections of NSCLC patients (**c**) and statistical analysis of IHC staining intensity and area (**d**). The expression of KLF5, GCN5, and GDF15 in NSCLC tumor tissues were increased compared to adjacent tissues (*N* = 185). **e** Quantification of plasma C5a in 40 NSCLC patients and 40 healthy volunteers by ELISA. Plasma C5a level of NSCLC patients was higher than control donors (**P < 0.01). **f**, **g** Representative result (**f**) and statistical analysis (**g**) for C5aR expression in NSCLC tissues by IHC. The C5aR expression was upregulated in NSCLC tumor tissues in comparison with adjacent tissues (*N* = 185)

First, we collected fresh tumor and adjacent tissues from 12 NSCLC patients, and total RNAs of every 4 tumor tissues were pooled. Subsequently, transcriptome sequencing was performed, and RNA-seq discovered that 411 genes in T1–T4, 542 genes in T5–T8, and 403 genes in T9–T12 were upregulated (≥2-fold) compared to paired adjacent tissues. Venn diagram showed that 86 genes were co-elevated in all samples (Fig. 1a). Meantime, gene ontology (GO) analysis found that 17.4% of theses 86 genes was involved in cell proliferation, growth, and maintenance (Fig. 1b). Next, we measured the mRNA levels of 12 proliferation-related genes in 40 NSCLC fresh tumor tissues. Real-time PCR exhibited that the mRNA levels of KLF5, HMGA1, FOXM1, EHF, HMGB3, SOX4, SOX9, GCN5, GDF15, MDK, TDGF1, and cyclin D1 were remarkably elevated, especially KLF5, GCN5, and GDF15 (Supplementary Figure 1a). Additionally, KLF5, GCN5 and GDF15 were positively correlated with each other (Supplementary Figure 1b). Subsequently, we observed marked increase of KLF5, GCN5, and GDF15 expression in 185 NSCLC tumor tissues by immunohistochemical (IHC) staining (Fig. 1c, d). To explore the association of C5a with NSCLC proliferation, we detected the plasma C5a of 40 NSCLC patients, and confirmed that C5a level was significantly upregulated (Fig. 1e). IHC also showed the high expression of C5aR (CD88) in tumor tissues of the above-mentioned 185 patients (Fig. 1f, g). Furthermore, we analyzed the correlation between KLF5, GCN5, GDF15, or C5aR expression and clinic-pathological parameters of the 185 NSCLC patients, and found that the expression of these proteins was tightly linked with the tumor size, lymph node metastasis, and TNM stage, but not related to tumor type, patient age and sex (Supplementary Tables 3 to 6). These findings suggest a certain connection between the expression of these genes and NSCLC tumorigenesis.

### C5a induces NSCLC cell proliferation and KLF5, GCN5, or GDF15 expression via binding to C5aR

**Fig. 2:**
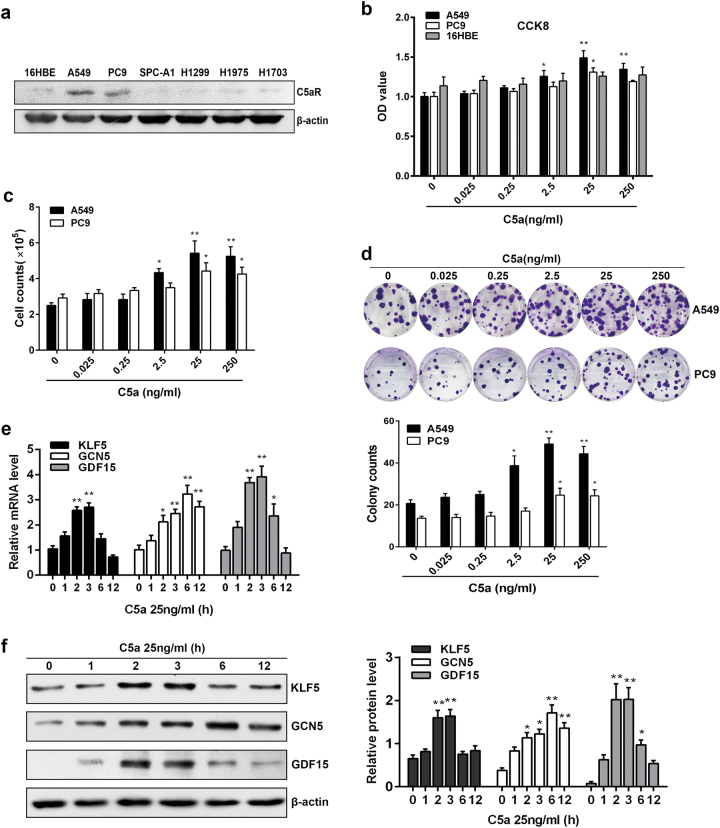
C5aR expression in human NSCLC cell lines as well as cell proliferation and KLF5, GCN5, GDF15 expression upon C5a stimulation. **a** IB assay showed that human C5aR was highly expressed in A549 and PC9 cell lines. **b**–**d** The proliferation of A549 and PC9 cells exposed to C5a (25 ng/ml and 250 ng/ml, especially 25 ng/ml) was notably increased by CCK8 (72 h, **b**), cell counting (7 days, **c**), and colony-forming assays (14 days, **d**). **e**, **f** Real-time PCR (**e**) and IB (**f**) exhibited that mRNA and protein of KLF5, GCN5, and GDF15 in A549 cells following 25 ng/ml C5a incubation started to increase from 1 h, and peaked at 2 h and 3 h (GCN5 peaked at 6 h). Representative photographs are manifested. Data are expressed as means ± S.E.M. from three independent experiments, *P < 0.05, **P < 0.01 vs. 0 ng/ml C5a

To investigate the mechanism of C5a-induced cell proliferation in NSCLC tumorigenesis, we detected C5aR in 6 human NSCLC cell lines, and found that C5aR expression was prominently upregulated in A549 and PC9 cell lines (Fig. 2a). Thereafter, we stimulated A549 and PC9 cells with recombinant C5a, and examined the cell proliferation by CCK8, cell counting and colony formation, and found that the cell viability, cell counts, and colony numbers were obviously increased when C5a concentration reached 25 or 250 ng/ml, particularly 25 ng/ml (Fig. 2b, d).

To verify that C5a can upregulate KLF5, GCN5, and GDF15 expression, A549 cells were stimulated with 25 ng/ml C5a, and then the mRNA and protein of the three genes were determined by real-time PCR and immunoblotting (IB). It displayed that mRNA and protein of these genes started to increase at 1 h upon C5a, peaked at 2 h and 3 h (GCN5 at 6 h, Fig. 2e, f).

To determine that the cell proliferation and KLF5, GCN5, or GDF15 expression in response to C5a are associated with C5a binding to C5aR, A549 cells were treated with DMEM, C5a, DMSO + C5a, C5aR inhibitor (C5aR-I, W54011) + C5a (C5aR-I + C5a), IgG + C5a, or Anti-C5a + C5a (the data of C5aR-I and Anti-C5a dose determination were not shown). We observed that C5aR-I and anti-C5a notably decreased cell viability (72 h), cell number (7d), colony number (14d) as well as KLF5, GCN5, and GDF15 expression (Supplementary Figure 2a–e). These indicate that the C5a-induced cell proliferation and KLF5, GCN5, or GDF15 expression require C5a-C5aR binding.

### Effect of KLF5, GCN5, or GDF15 expression on C5a-driving cell proliferation including their relationship with each other

**Fig. 3:**
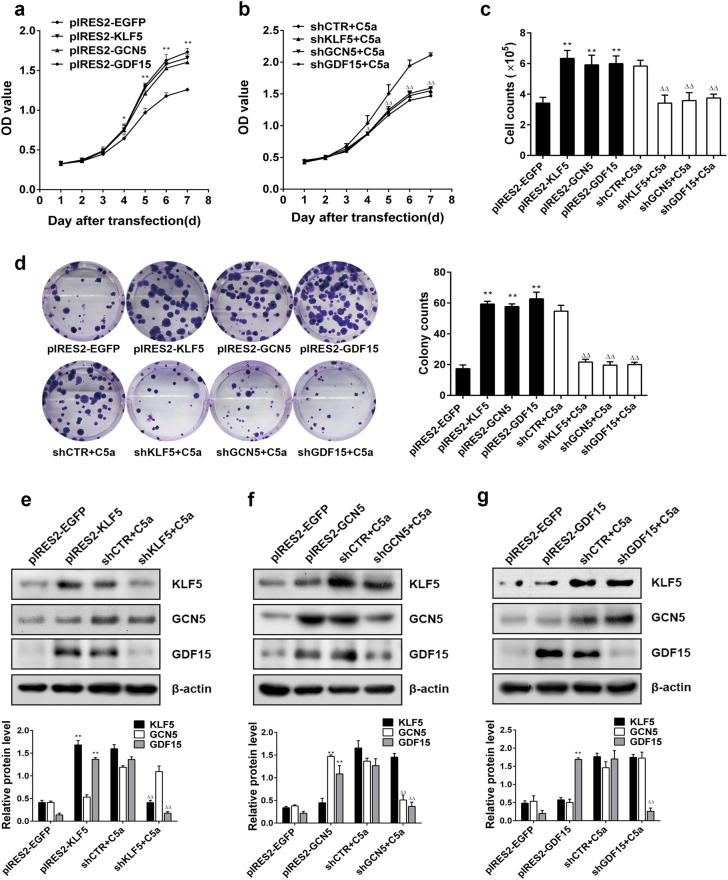
Roles of KLF, GCN5, and GDF15 expression in A549 cell proliferation and their relationships of the three genes. **a**–**d** CCK8 (**a**, **b**), cell counting (**c**), colony formation (**d**) assays showed that the proliferation of A549 cells transfected with KLF5, GCN5, and GDF15 overexpression plasmids for 48 h or with the interference plasmids for 48 h followed with C5a for 3 h were increased or decreased (pIRES2-EGFP or shCTR + C5a as control). **e**–**g** IB results exhibited that overexpression of KLF5 or GCN5 gene in A549 cells could increase GDF15 expression, and the C5a-induced GDF15 upregulation was repressed by KLF5 or GCN5 knockdown, but the change of GDF15 expression did not cause marked variation of KLF5 and GCN5 levels. All data represent means ± S.E.M. of three independent assays, and representative pictures are presented. *P < 0.05, **P < 0.01 vs. pIRES2-EGFP; ^△△^*P* < 0.01 vs. shCTR + C5a

Given that the KLF5, GCN5, and GDF15 expression was earlier than cell proliferation in response to C5a, we suspected that these proteins might regulate cell proliferation. To elucidate the role and relationship of KLF5, GCN5, and GDF15, we constructed corresponding overexpression or interference plasmids (Supplementary Figure 3a and b), and confirmed that KLF5, GCN5, or GDF15 overexpression improved A549 cell proliferation, while knockdown of them inhibited the C5a-triggered cell proliferation (Fig. 3a–d), suggesting that the C5a-induced upregulation of these genes has an important role in A549 cell proliferation.

When ascertained the up- and downstream relationship within the three genes, we observed that while overexpressing or silencing KLF5 and GCN5 could increase or decrease GDF15 expression (Fig. 3e, f), the change of GDF15 did not affect KLF5 or GCN5 expression (Fig. 3g), implicating that GDF15 is in the downstream of KLF5 and GCN5.

### C5a, KLF5, and GCN5 promote GDF15 gene transcription in A549 cells

Since KLF5 and GCN5 could increase GDF15 expression (Fig. 3e, f), further experiments were done to determine whether KLF5 or GCN5 could directly trigger GDF15 gene transcription. Firstly, we demonstrated that GDF15 full-length (FL) promoter (−2078 to +103 nt) activity was greatly boosted by C5a at 1 h, peaked at 2 h and 3 h (Supplementary Figure 4a), and descended markedly when cells were pretreated with C5aR-I or Anti-C5a (Supplementary Figure 4b). Importantly, overexpression or knockdown of KLF5 and GCN5 upregulated or downregulated GDF15 promoter activity (Supplementary Figure 4c). These data hint that C5a-enhanced KLF5 and GCN5 expression can directly trigger and augment GDF15 gene transcription.

### KLF5 and GCN5 improve GDF15 gene transcription via forming a complex on GDF15 promoter in A549 cells upon C5a exposure

**Fig. 4:**
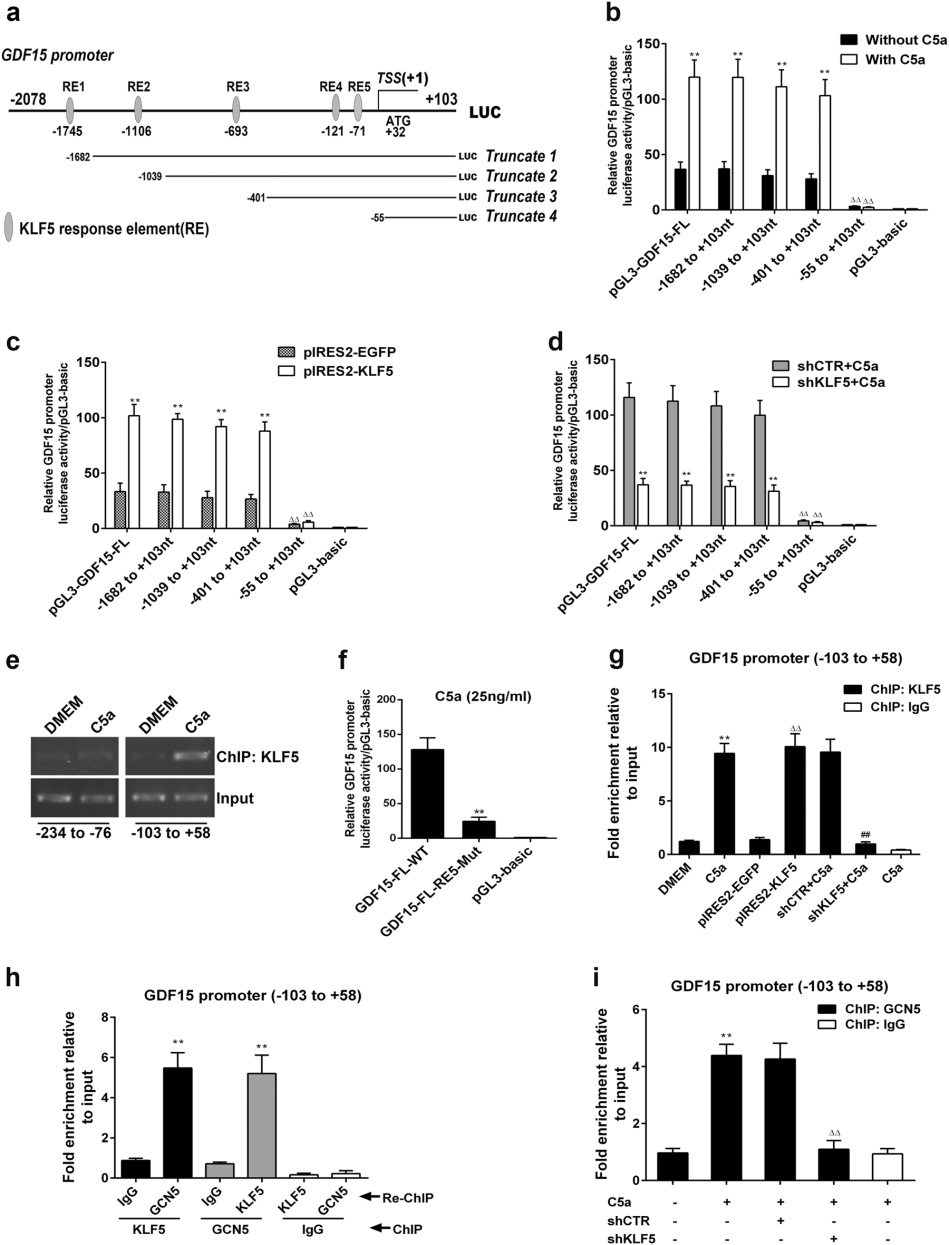
Identification of KLF5-binding site to GDF15 promoter and effect of C5a stimulation, KLF5 overexpression or knockdown on KLF5 and GCN5 binding to the promoter. **a** Schematic drawing of GDF15 promoter region (−2078 to +103 nt). On the basis of five KLF5 response elements (RE1 to RE5) predicted by JASPAR, the reporter plasmids of GDF15-FL (−2078 to +103 nt), truncate 1 (−1682 to +103 nt), truncate 2 (−1039 to +103 nt), truncate 3 (−401 to +103 nt) and truncate 4 (−55 to +103 nt) promoters were constructed. **b**, **c** A549 cells were transfected with the GDF15-FL or different truncated GDF15 promoters and then treated with C5a stimulation for 3 h (**b**) or pIRES2-KLF5 transfection for 48 h (**c**). Luciferase reporter analysis showed that the GDF15 promoter activity was markedly increased upon C5a or KLF5 overexpression (**P < 0.01 vs. without C5a or pIRES2-EGFP), except for truncate 4 group, and the promoter activity in truncate 4 group was dramatically lower than that in GDF15-FL, truncate 1, 2, and 3 groups (^△△^*P* < 0.01). **d** A549 cells were co-transfected with the above-mentioned GDF15 promoter plasmids and shKLF5 or shCTR plasmids. The GDF15 promoter activity was suppressed when KLF5 was silenced, even in the present of C5a stimulation (**P < 0.01), apart from truncate 4 group, and the activity in truncate 4 group reduced significantly compared with GDF15-FL, truncate 1, 2, and 3 groups (^△△^*P* < 0.01). **e** A549 cells were treated with C5a, and then ChIP assay was performed with KLF5 antibody. The purified DNA was amplified by PCR for GDF15 promoter regions of −234 to −76 nt (containing RE4) and −103 to +58 nt (containing RE5). It showed that C5a greatly increased the level of KLF5 binding to GDF15 promoter (−103 to +58 nt). **f** A549 cells were transfected with the GDF15-FL or GDF15-RE5-Mut reporter plasmid and stimulated by C5a for 3 h. Luciferase assay showed the mutation of RE5 could markedly inhibit the C5a-induced activation of GDF15 promoter. **g** ChIP assay using anti-KLF5 and real-time PCR revealed that the content of KLF5 binding to the region of GDF15 promoter elevated in both C5a and pIRES2-KLF5 groups (**P < 0.01 vs. DMEM; ^△△^*P* < 0.01 vs. pIRES2-EGFP), and reduced in shKLF5 + C5a group (^##^*P* < 0.01 vs. shCTR + C5a). **h** A549 cells were stimulated by C5a for 3 h. Anti-KLF5 and GCN5 abs were used to perform ChIP and Re-ChIP assays. It showed that the KLF5 could bind to GDF15 promoter (−103 to +58 nt) together with GCN5 (**P < 0.01 vs. IgG). **i** ChIP performed by using the anti-GCN5 ab exhibited that the binding of GCN5 to GDF15 promoter (−103 to +58 nt) increased with C5a stimulation (**P < 0.01 vs. DMEM), but diminished by KLF5 interference (^△△^*P* < 0.01 vs. shCTR + C5a). Representative photographs are displayed. All data represent means ± S.E.M. of three independent experiments

Because KLF5 and GCN5 can enhance GDF15 promoter activity, the mechanism in C5a-induced GDF15 gene transcription requires to be explored. First, five KLF5-binding elements (RE1 to RE5) on GDF15 promoter were predicted by JASPAR software (Fig. 4a). Then, the plasmids of four truncated GDF15 promoter fragments (truncate 1: −1682 to +103 nt, truncate 2: −1039 to +103 nt, truncate 3: −401 to +103 nt and truncate 4: −55 to +103 nt) were constructed. Further experiments showed that the activity of truncate 4 promoter was acutely reduced compared with FL or other truncated promoters, and did not change after C5a stimulation, KLF5 overexpression or KLF5 knockdown (with C5a incubation) in A549 cells (Fig. 4b–d). These results implicate the effective KLF5-binding sites on GDF15 promoter might locate within −401 to −55 nt and may be RE4 (−122 to −113 nt) and/or RE5 (−71 to 62 nt).

To further identify the exact site of KLF5 binding to GDF15 promoter, chromatin immunoprecipitation (ChIP)-real-time PCR was performed and found that KLF5 could bind to the region (−103 to +58 nt) of GDF15 promoter, which was eminently augmented by C5a (Fig. 4e), demonstrating that this region of GDF15 promoter contains KLF5-binding element (probably RE5). Expectedly, the mutation of RE5 (AGGGCGGGAC to AAAAAAAAAA) could significantly reduce the C5a-triggered activation of the GDF15-FL promoter (Fig. 4f).

Subsequently, we continued to assess the enrichment of KLF5 on GDF15 promoter (−103 to +58 nt) in KLF5 overexpression or knockdown. It displayed that the binding level was greatly increased after KLF5 overexpression, and markedly decreased when KLF5 was silenced even in the present of C5a (Fig. 4g), indicating that KLF5 can directly bind to GDF15 promoter and improve GDF15 gene transcription.

GCN5 is reported to be a co-activator that can be recruited to promoter and enhanced gene transcription [17, 18]. Hence, to clarify whether C5a can induce GCN5 to bind to GDF15 promoter, ChIP and Re-ChIP assays were performed and showed that GCN5 and KLF5 could bind to GDF15 promoter (−103 to +58 nt) through forming a complex (Fig. 4h). During this process, there was a notable reduction of GCN5 binding on GDF15 promoter when interfering KLF5 expression (Fig. 4i). These data reveal that C5a can lead to KLF5 and GCN5 forming a complex and binding to GDF15 promoter in a KLF5-dependent manner, and finally enhance GDF15 gene transcription.

### KLF5 acetylation by GCN5 promotes GDF15 gene transcription through elevating KLF5 binding to GDF15 promoter

**Fig. 5:**
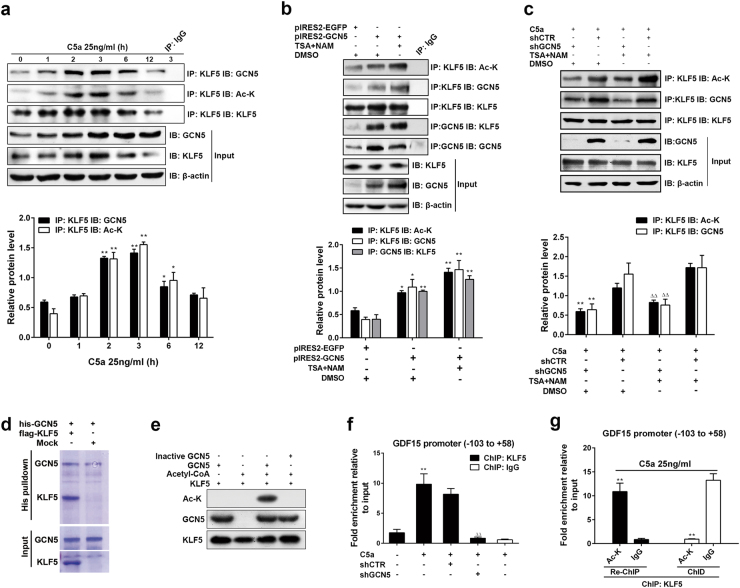
The GCN5–KLF5 interaction and KLF5 acetylation upon C5a stimulation or change of GCN5, and the effect of KLF5 acetylation by GCN5 on binding to GDF15 promoter. **a** IP-IB was done by using abs against KLF5 and GCN5. The related level of KLF5 acetylation and GCN5–KLF5 binding was adjusted to precipitated total KLF5. It showed that C5a could enhance GCN5–KLF5 binding and KLF5 acetylation in A549 cells at 2, 3, and 6 h (*P < 0.05, **P < 0.01 vs. 0 h). **b** A549 cells transfected with pIRES2-GCN5 for 48 h were then treated with (or without) trichostatin A (TSA, 1 μM) and nicotinamide (NAM, 5 μM) for 6 h. By IP, the level of GCN5–KLF5 combination or KLF5 acetylation was upregulated after GCN5 overexpression, which was more significant in the present of TSA and NAM (*P < 0.05, **P < 0.01 vs. pIRES2-EGFP + DMSO). **c** A549 cells transfected with shGCN5 for 48 h were treated with TSA and NAM for 6 h, followed by C5a for 3 h. IP exhibited a notable decrease of GCN5–KLF5 binding and KLF5 acetylation, even with TSA and NAM treatment (**P < 0.01 vs. shCTR + C5a + DMSO, ^△△^*P* < 0.01 vs. shCTR + C5a + TSA + NAM). **d** The his-GCN5 protein was bound to the cobalt-coated beads, followed by purified flag-KLF5 incubation. The pulldown mixture was subjected to SDS-PAGE and coomassie blue staining. The result showed GCN5 could directly bind to KLF5. Mock: elution from anti-flag affinity gel incubated with the cell lysate without flag-KLF5. **e** Purified KLF5 was incubated with acetyl-CoA and active or inactive GCN5. The acetylation of KLF5 was measured by IB using the acetylated lysine antibody. It showed that the active GCN5 but not inactive GCN5 could acetylated KLF5. **f** The ab against KLF5 was employed to perform ChIP assay, and real-time PCR displayed that the binding of KLF5 on GDF15 promoter region (−103 to + 58 nt) in C5a group was higher than DMEM group (**P < 0.01), but the binding level in shGCN5 + C5a group markedly dropped compared with shCTR + C5a group (^△△^*P* < 0.01). **g** A549 cells were exposed to C5a stimulation for 3 h. The ChIP assay was performed with anti-KLF5, followed by the Re-ChIP assay with anti-Ac-K. Purified DNA in the precipitation and supernatant were then amplified by real-time PCR to detect GDF15 promoter fragment. Re-ChIP proved the enrichment of acetylated KLF5 in GDF15 promoter was greatly increased, while ChID showed that GDF15 promoter fragment was significantly diminished in the supernatant after Re-ChIP (**P < 0.01 vs. IgG). Representative photographs are showed. Data are expressed as means ± S.E.M. from at least three independent experiments

As we know, GCN5 is a acetyltransferase which can modify protein lysine [17, 18], but whether GCN5 can acetylate KLF5 and whether acetylated KLF5 can affect GDF15 transcription remain unknown. To solve these problems, IP was done and exhibited an interaction between GCN5 and KLF5 as well as an upregulation of KLF5 acetylation following C5a stimulation (Fig. 5a). Additionally, the level of GCN5–KLF5 binding and KLF5 acetylation in A549 cells with C5aR-I or Anti-C5a treatment was markedly downregulated (Supplementary Figure 5), implying that C5a stimulation can result in GCN5–KLF5 combination and KLF5 acetylation via C5a-C5aR. Furthermore, the GCN5–KLF5 binding and KLF5 acetylation were significantly increased by GCN5 overexpression (Fig. 5b), while C5a-induced GCN5–KLF5 binding and KLF5 acetylation remarkably reduced when GCN5 was silenced, even in the present of deacetylase inhibitor trichostatin A (TSA) and nicotinamide (NAM, Fig. 5c). Meantime, the direct binding of purified recombinant KLF5 and GCN5, or the KLF5 acetylation modified by GCN5 was also confirmed by pulldown assay (Fig. 5d) or in vitro acetylation assay (Fig. 5e). These suggest that GCN5 bears a great responsibility in C5a-induced KLF5 acetylation.

To study the impact of GCN5 and acetylated KLF5 on GDF15 gene transcription, we performed ChIP, Re-ChIP, and ChID assays and found that the binding of KLF5 on GDF15 promoter (−103 to +58 nt) was diminished after GCN5 silence (Fig. 5f), and only acetylated KLF5 could greatly bind to the region of GDF15 promoter (Fig. 5g). Therefore, we conclude that the GDF15 gene transcription in C5a-stimulated A549 cells was attributed to KLF5 acetylation by GCN5.

### KLF5 acetylation at lysine 335 and lysine 391 via GCN5 regulates GDF15 gene transcription, expression, and cell proliferation triggered by C5a

**Fig. 6:**
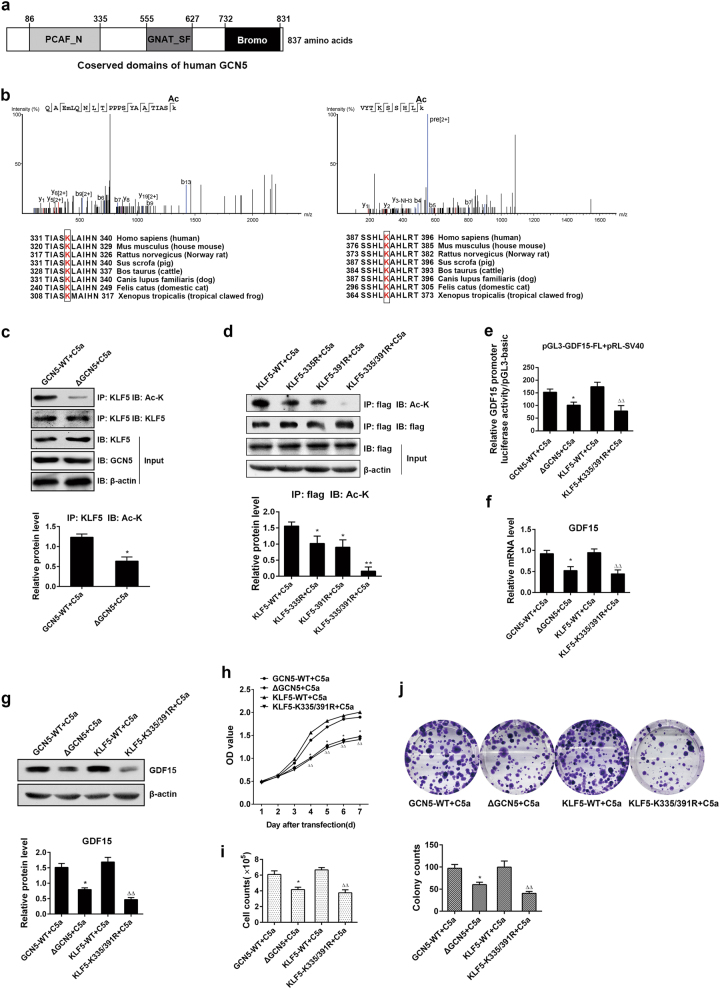
Roles of KLF5 acetylation by GCN5 in C5a-induced GDF15 gene transcription, expression and A549 cell proliferation. **a** Schematic drawing of conserved domains in human GCN5 protein. The GNAT-SF domain has HAT activity. **b** A549 cells were overexpressed with GCN5 and flag-KLF5, and the purified KLF5 was analyzed by mass spectrometry. It exhibited that lysine 335 (K335) and lysine 391 (K391) of KLF5 protein were acetylated. Alignment of KLF5 amino acids sequence among different species showed K335 and K391 are highly conserved. **c**, **d** A549 cells transfected with the vectors expressing GCN5-WT, △GCN5 (GCN5 lack of GNAT-SF), flag-tagged KLF5-WT, KLF5-335R, KLF5-391R (lysine to arginine mutant), and KLF5-335/391R (double mutation of lysine 335 and 391). The KLF5 acetylation level detected by IP with abs against KLF5 or flag and IB with anti-Ac-K notably decreased in △GCN5 + C5a group compared with GCN5-WT + C5a (**c**), and was markedly lower in KLF5–K335R + C5a, KLF5-391R + C5a and KLF5–K335/391R + C5a groups than in KLF5-WT + C5a group (**d**), particularly more significantly in KLF5–K335/391R + C5a group (*P < 0.05, **P < 0.01). **e** Luciferase reporter analysis displayed that GDF15 promoter (FL) activity in △GCN5 + C5a and KLF5–K335/391R + C5a groups was decreased (*P < 0.05 vs. GCN5-WT + C5a; ^△△^*P* < 0.01 vs. KLF5-WT + C5a). **f**, **g** Real-time PCR (**f**) and IB (**g**) assays manifested that GDF15 mRNA and protein in △GCN5 + C5a and KLF5–K335/391R + C5a groups were diminished (*P < 0.05 vs. GCN5-WT + C5a; ^△△^*P* < 0.01 vs. KLF5-WT + C5a). **h**–**j** CCK8 (**h**), cell counting (**i**), and colony formation (**j**) assays showed the proliferation of A549 cells in △GCN5 + C5a and KLF5–K335/391R + C5a groups was lessened (*P < 0.05 vs. GCN5-WT + C5a; ^△△^*P* < 0.01 vs. KLF5-WT + C5a). Means ± S.E.M. is presented, and results are representative of three independent experiments. Representative photographs are exhibited

To further define the role of KLF5 acetylation by GCN5 in GDF15 gene transcription, expression and cell proliferation mediated by C5a, we transfected A549 cells with a HAT (GNAT-SF) domain (Fig. 6a)-deleted GCN5 mutant (△GCN5) followed with C5a treatment for 3 h and uncovered a significant decrease of KLF5 acetylation compared with wild-type (WT) GCN5 (Fig. 6c). On the other hand, according to the mass spectrometry result that KLF5 was acetylated by GCN5 at two highly conserved acetylation sites, lysine 335 (K335) and lysine 391 (K391, Fig. 6b), we mutated the two sites separately or together by changing lysine (K) to non-acetylatable arginine (R). It showed that KLF5 acetylation was remarkably downregulated when K335 or K391 was mutated, and the downregulation was even more eminent upon the two sites mutation (Fig. 6d). Meanwhile, we observed that the HAT domain-deletion of GCN5 and double mutation of KLF5 at K335 and K391 could markedly decrease GDF15 promoter activity, GDF15 expression (Fig. 6e–g), and A549 cells proliferation (Fig. 6h–j). Besides, the exogenous KLF5 acetylation by GCN5 in A549 cells exposed to C5a was also reduced in the condition of previous-mentioned mutations, and so were the promoter activity and expression of GDF15, as well as A549 cell proliferation (Supplementary Figure 6a–f). Together, these results implicate that the KLF5–K335 and K391 acetylation by GCN5 has a pivotal role in mediating GDF15 gene transcription, expression and A549 cell proliferation.

### Effect of silencing KLF5, GCN5, or GDF15 gene on mice A549 xenograft tumor growth and proliferation-related protein expression

**Fig. 7:**
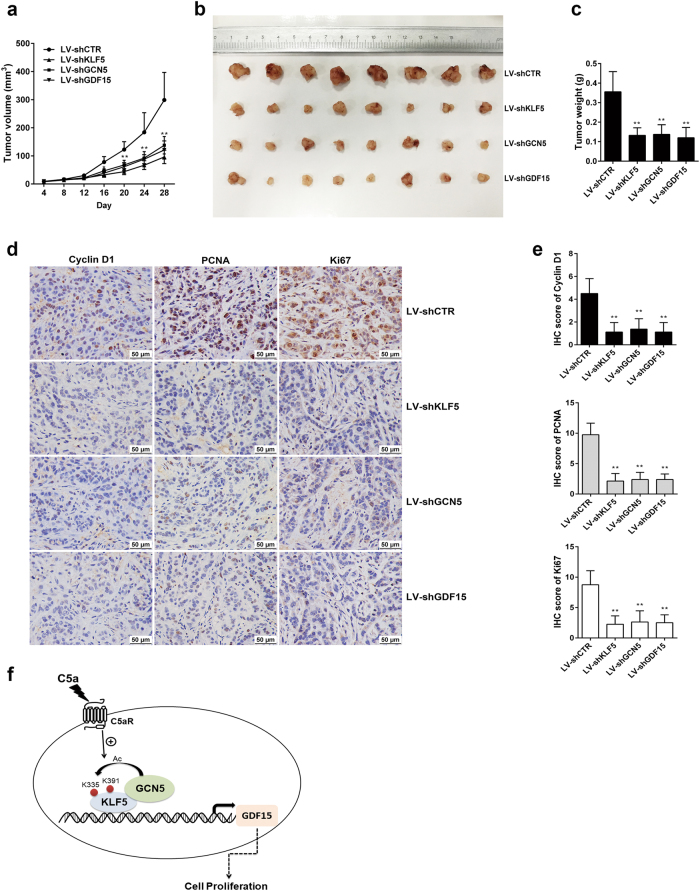
Effect of KLF5, GCN5, or GDF15 on tumor growth and cell proliferation in BALB/c nude mouse model. **a**–**c** A549 cells (5 × 10^6^) infected stably with corresponding LV-shRNAs were inoculated subcutaneously into the axilla of 5-week-old BALB/c nude mice (*n* = 8) to generate lung adenocarcinoma-bearing mouse models. The volume of xenograft tumors was monitored every 4 days, and tumors were photographed and weighed on 28d after inoculation. The results showed that in comparison with the mice of LV-shCTR (control) group, the volume (**a**), size (**b**), and weight (**c**) in the mice bearing LV-shKLF5, LV-shGCN5, or LV-shGDF15 xenograft tumors were notably reduced (**P < 0.01 vs. LV-shCTR). **d**, **e** Representative images of IHC staining for cyclin D1, PCNA, and Ki67 in xenograft tissues (**d**) and score analysis of IHC staining intensity and area (**e**). The expression of cyclin D1, PCNA, and Ki67 was markedly downregulated in LV-shKLF5, LV-shGCN5, or LV-shGDF15 group (**P < 0.01 vs. LV-shCTR). **f** The putative scheme for the molecular mechanism of NSCLC proliferation induced by C5a. In response to C5a, KLF5, GCN5, and GDF15 are all overexpressed, and the upregulated KLF5 can recruit GCN5 and bind to the GDF15 promoter as a complex. In addition, GCN5 can acetylate KLF5 at lysine 335 and lysine 391 to strengthen the combination of KLF5 and GDF15 promoter, and further augment GDF15 gene transcription and expression, leading to NSCLC cell proliferation

Since we already found the pro-proliferative effect of KLF5, GCN5, and GDF15 in vitro, we wondered the function of these genes in vivo. To explore this, A549 cells infected stably with lentiviruses carrying shCTR, shKLF5, shGCN5, or shGDF15 (Supplementary Figure 7a and b) were injected subcutaneously into BALB/c nude mice. Thereafter, the xenograft growth was monitored for up to 28 days. The results showed that the volume, size, and weight of LV-shKLF5, LV-shGCN5, or LV-shGDF15 xenografts were prominently less than that of LV-shCTR tumors (Fig. 7a–c). Similarly, a marked suppression of cyclin D1, PCNA and Ki67 expression was also displayed in above-mentioned xenografts (Fig. 7d, e), although no histomorphological differences were seen in all xenografts (Supplementary Figure 7c). Additionally, GDF15 protein was significantly downregulated in LV-shKLF5 and LV-shGCN5 xenograft, and KLF5 acetylation was notably reduced in LV-shGCN5 xenograft (Supplementary Figure 7d and e). The findings in vivo confirmed again that KLF5, GCN5, and GDF15 expression could accelerate NSCLC growth and cell proliferation.

## Discussion

Inflammation is a contributor to NSCLC progression [10–12], and C5a can cause tumor cell proliferation [8, 9, 28, 29]. However, how C5a affects tumor cell proliferation in NSCLC still remains obscure.

In this study, we scrutinized some proliferation-related genes, and found that KLF5, GCN5, and GDF15 including C5a and C5aR levels were elevated in the majority of NSCLC patients. We also discovered that KLF5, GCN5, GDF15, and C5aR expression was closely correlated with the tumor size, lymph node metastasis, and TNM stage of NSCLC, indicating that these proteins may have an important function in NSCLC tumorigenesis.

Reportedly, complement could be activated in tumor tissues [2, 30, 31], and lung cancer cells could also generate C5a [3], creating a microenvironment for NSCLC growth and proliferation [11, 12, 30]. Thus, to explore the effect of C5a on NSCLC cell proliferation, the C5aR expression in multiple NSCLC cell lines was detected and the upregulation of C5aR in A549 and PC9 cells was found. Subsequent experiments exhibited that the proliferation of A549 and PC9 cells increased upon C5a stimulation, particularly that of A549 cells. Further studies discovered that the KLF5, GCN5, and GDF15 expression in C5a-treated A549 cells was upregulated and blocking C5aR or neutralizing C5a could remarkably repress the expression of these three proteins and cell proliferation, suggesting that C5a possesses the capacity of promoting these genes’ expression and cell proliferation via binding to C5aR. Notably, some pro-inflammatory factors such as IL-1 and IL-6 can upregulate C5aR expression during the process of other diseases [32, 33]. These factors, which also exist in NSCLC microenvironment [10], might elevate C5aR expression in NSCLC cells, and finally promoting NSCLC development and progression together with C5a.

Recently, researches have confirmed that KLF5 and GCN5 are overexpressed in many cancers [16, 17], and could regulate tumor biological processes [34, 35] as a transcription factor and a transcriptional co-activator respectively. Many studies also consider that GDF15 is a contributor to the development and progression of some malignant tumors [20, 21, 36–38], although several documents have reported that retinoid-related molecule or anti-inflammatory drug can induce tumor cell apoptosis via GDF15 [39–41]. Bruzzese F^20^ points out that the pro- and anti-tumor activities of GDF15 are highly dependent on the cellular and microenvironmental context. Our present studies revealed that overexpression or knockdown of KLF5, GCN5, and GDF15 genes in A549 cells could promote or inhibit cell proliferation, implicating that these proteins have a pro-proliferation function. Meanwhile, we found that overexpression or knockdown of KLF5 and GCN5 upregulated or downregulated GDF15 expression, but overexpressing or silencing GDF15 gene did not alter KLF5 and GCN5 expression, hinting that GDF15 is a downstream gene of KLF5 and GCN5 in C5a-governing proliferation.

Emerging evidence has shown that GDF15 overexpression can facilitate tumor cells proliferation, e.g., ovarian cancer [37], breast cancer [21], and esophageal squamous cell carcinomas [38]. Our data proved that GDF15 promoter activity was upregulated by C5a stimulation. Correspondingly, overexpression or knockdown of KLF5 and GCN5 also elevated or reduced GDF15 promoter activity. These findings suggest that C5a-induced proliferation is due to the increase of KLF5, GCN5, and GDF15 expression, and KLF5 and GCN5 overexpression has an improving role in GDF15 gene transcription.

To date, KLF5 binding to GC boxes on gene promoters and regulating gene transcription have been reported [14, 42]. Moreover, it is also demonstrated that GCN5 can amplify transcription factor effect [17, 18, 43]. The luciferase assay revealed that KLF5 expression augmented GDF15 promoter activity, and the KLF5-binding elements were located within −401 to −55 nt of GDF15 promoter. Furthermore, we discovered for the first time that KLF5 could bind to the region −103 to +58 nt of GDF15 promoter, containing the response element AGGGCGGGAC, and that C5a could cause the interaction between KLF5 and GCN5, leading them to form a complex in a KLF5-dependent manner, and finally promote GDF15 gene transcription in A549 cells.

Protein function is often regulated by posttranslational modifications such as acetylation [18, 44]. As GCN5 is an acetyltransferase modifying histone or non-histone protein such as transcription factor [43], KLF5 acetylation in GDF15 gene transcription and NSCLC cell proliferation need to be illustrated. In the study, we first disclosed that KLF5 could be acetylated by GCN5 at lysine 335 and lysine 391 via mass spectrometry. Notably, only acetylated KLF5 could bind to GDF15 promoter, and failure in KLF5 acetylation in A549 cells transfected with ΔGCN5 or KLF5–K335R and K391R mutants not only prevented KLF5–GCN5 complex formation, but also lessened the binding of KLF5 to GDF15 promoter. These imply that KLF5 acetylation is essential for KLF5 recruitment to GDF15 promoter and GDF15 gene transcription, expression as well as A549 cell proliferation.

In vivo, we demonstrated delayed growth and proliferation in nude mice xenograft tumors created by inoculating A549 cells stably infected with LV-shKLF5, LV-shGCN5, or LV-shGDF15, indicating that KLF5, GCN5 and GDF15 are necessary in the maintenance of NSCLC growth.

In conclusion, our studies revealed that KLF5, GCN5, GDF15, C5a, and C5aR were increased in the samples of NSCLC patients, and in vitro C5a could induce KLF5, GCN5, or GDF15 expression, promoting A549 cell proliferation. The C5a-elevated KLF5 could recruit GCN5, forming a complex which bound to GDF15 promoter in a KLF5-dependent manner and enhancing GDF15 gene transcription. In this process, KLF5 could be acetylated by GCN5 at lysine 335 and lysine 391, facilitating the binding of KLF5 with GDF15 promoter and upregulating GDF15 expression as well as A549 cell proliferation (Fig. 7d). Besides, GCN5, KLF5, or GDF15 silence reduced cell proliferation in vitro and xenograft tumor growth in vivo. Together, these findings provide clues to the mechanism of C5a-induced NSCLC proliferation, which may also provide targets for human NSCLC therapy.

## Materials and methods

### Human specimens and animals

The fresh NSCLC tissues and the paired adjacent tissues of 52 patients were collected during the surgery from the First Affiliated Hospital of Nanjing Medical University and Jiangsu Cancer Hospital. Paraffin-embedded NSCLC tumor tissues (*N* = 185) were provided by National Engineering Center for BioChips or collected from the First Affiliated Hospital of Nanjing Medical University. All samples were from patients who underwent surgical resection without preoperative chemotherapy or radiotherapy. Patients in any other disease conditions (infections, allergies, or other inflammatory diseases and cancers) were excluded. The study was also approved by the Ethics Committee of Nanjing Medical University and informed consent was obtained from all patients participating in this research prior to the experiment. Female BALB/c nude mice (5-week-old) were from Vital River Laboratory Animal Technology Co. Ltd (Beijing, China). Mice were maintained in animal facilities under pathogen-free conditions, and the animal study was approved by the Animal Ethical and Welfare Committee (AEWC) of Nanjing Medical University.

### Cell lines and reagents

The human NSCLC cell lines, A549, H1299, H1975, and H1703 were obtained from the American Type Culture Collection (ATCC). The PC9 and SPC-A1 cells were from the European Collection of Authenticated Cell Cultures (ECACC) and the Institute of Biochemistry and Cell Biology of the Chinese Academy of Science, respectively. The human bronchial epithelial cell line (16HBE) was provided by Dr Gruenert (California Pacific Medical Center, San Francisco, CA, USA). All of the cell lines were authenticated by short tandem repeat profiling and free of mycoplasma contamination. The recombinant human C5a was from R&D systems (Tustin, CA, USA), and C5aR antagonist W54011 was provided by Merck Millipore (Darmstadt, Germany). The antibodies (Abs) against C5a (ab135187), GDF15 (ab180929) and cyclin D1 (ab134175) were supplied by Abcam (Cambridge, UK). The KLF5 (sc-22797×) and GCN5 (sc-365321×) Abs were from Santa Cruz Biotechnology (Santa Cruz, CA, USA). The Abs of acetylated lysine (Ac-K, #9441 or #9681), anti-PCNA (#13110) and chromatin immunoprecipitation (ChIP) kit were from Cell Signaling Technology (Danvers, MA, USA). The Abs against C5aR (P21730) and Ki67 (P46013) were from Bioworld Technology (St. Louis Park, MN, USA). DAB substrate kit, C5a ELISA kit and his-tag isolation & pulldown dynabeads were from Thermo Fisher (Waltham, MA, USA). Dual-luciferase reporter assay system kit was purchased from Promega (Madison, WI, USA). The recombinant his-tagged GCN5 was from Cayman Chemical (Ann Arbor, MI, USA), and the acetyl-CoA was from Solarbio (Beijing, China). The anti-flag immunoaffinity resin was supplied by Sigma-Aldrich (St. Louis, MS, USA) and QuikChange II site-directed mutagenesis kit was from Agilent (Santa Clara, CA, USA).

### Transcriptome deep sequencing

Total RNA from 12 pairs of NSCLC tumor and adjacent tissues were isolated. The samples were generated by mixing the RNAs from 4 patients and the sequencing library of each mixing sample was prepared. The RNA library was then sequenced on Illummina HiSeq 2500 [45].

### Quantitative real-time PCR

Total RNA of tissues or cells was prepared, and the cDNA was then generated. Real-time PCR experiment was performed. The primers for real-time PCR are shown in Supplementary Table 1. The results were normalized to the β-actin expression and analyzed by using the 2^−ΔΔCt^ method [22, 25].

### Immunohistochemical staining

The sections of patient NSCLC tumor and mouse xenograft tumor were incubated with Abs against KLF5, GCN5, GDF15, C5aR, cyclin D1, PCNA, and Ki67. The IHC was scored according to staining intensity. For each sample, 500 cells from five randomly chosen fields were counted. For IHC intensity scoring: negative = 0, weak = 1, moderate = 2, strong = 3. For staining area scoring: 0% = 0, 1–25% = 1, 26–50% = 2, 51–75% = 3 and 76–100% = 4. These scores were multiplied to produce the final score: 0–1, negative expression; 2–4, weak positive expression; 6–12, strong positive expression [46].

### ELISA detection

The plasma from 40 patients and 40 healthy individuals were collected from the First Affiliated Hospital of Nanjing Medical University. The C5a concentration was measured using ELISA kit according to manufacturer’s instructions.

### Plasmids construction

The plasmids of pIRES2-KLF5, pIRES2-GCN5, pIRES2-GDF15, flag-KLF5 were constructed by inserting complete open reading frames (ORF) of human KLF5 (NM_001730.4), GCN5 (NM_021078.2), and GDF15 (NM_004864.2) gene into pIRES2-EGFP vector. Primer sequences are listed in Supplementary Table 2. Then, shKLF5, shGCN5, and shGDF15 plasmids were constructed using pGpU6/GFP/Neo vector and the most effective shRNA was chosen for further experiment. The shRNA targeted sequences are as follows: shKLF5, TGCTGTTCCGCAGACTGCAGTGAAA; shGCN5, GAGGACGTGGCTACCTACAAGGTCA; shGDF15, CCGGATACTCACGCCAGAAGT. Moreover, the mutant GCN5 (ΔGCN5) which deleted acetyltransferase domain and KLF5 of which lysine 335 (K335), lysine 391 (K391), or both two lysine sites (K335/391) were mutated to non-acetylatable arginine (R) were created.

### Lentiviral shRNA packing

The plasmids of lentiviral (LV)-shKLF5, LV-shGCN5, and LV-shGDF15 were prepared by Genechem (Shanghai, China). The shRNA sequences used to silence corresponding genes are the same as previous-described most effective shRNAs against the three genes.

### Cell culture and transfection

NSCLC tumor cell lines were cultured in DMEM with 10% FBS. For transient transfection, cells were cultured overnight, and the mixture of plasmid and transfection reagent was added, incubated for 48 h. For stable transfection, 2 × 10^5^ A549 cells were incubated with LV at the titer of 4 × 10^6^ TU/ml for 48 h and then challenged by puromycin selection. The transfection efficiency was estimated by GFP and corresponding protein expression.

### Immunoprecipitation (IP) assay

Protein extracted from A549 cells and tissues was incubated with protein G-Sepharose beads and pre-immune IgG. After centrifugation, the supernatant was collected and incubated with corresponding Abs. The antigen–antibody complex was precipitated with protein G agarose beads, and IB assay for detecting protein and acetylated lysine was performed [25].

### Immunoblotting (IB) analysis

The whole-cell lysates (WCL) were electrophoresed. The blots were probed with primary Abs. Thereafter, the blots were incubated with corresponding secondary Abs, and exposed using regular X-Ray film or Amersham imager 600 (GE, Boston, MA, USA) [22, 25].

### Luciferase reporter experiment

GDF15 full-length (GDF15-FL) promoter plasmids were constructed by inserting GDF15 promoter fragment (−2068 to +103 nt) into pGL3-basic vector. The promoter deletion fragments (truncate 1: −1682 to +103 nt, truncate 2: −1039 to +103 nt, truncate 3: −401 to +103 nt and truncate 4: −55 to +103 nt) were cloned into the same vector. Specific primers for promoter luciferase assays are listed in Supplementary Table 2. The GDF15-FL-RE5-Mut plasmid was constructed by mutating the KLF5 response element 5 on GDF15-FL (RE5, −71 to 62 nt, AGGGCGGGAC) to the AAAAAAAAAA sequence. The pGL3-GDF15-FL and truncated 1 to 4 plasmids were transfected into A549 cells. The promoter activity was measured by dual-luciferase reporter reagent [47].

### ChIP, Re-ChIP, and ChID assays

Chromatin immunoprecipitation (ChIP) assay was performed using ChIP-validated Abs and ChIP-grade protein G agarose beads. For Re-ChIP, the primary ChIP material was released from the agaroses and subjected to second round of immunoprecipitation using corresponding Abs. For chromatin immunodepletion (ChID), ChIP material isolated from the agaroses was immunoprecipitated again with an anti-Ac-K Ab, and GDF15 promoter fragment in the supernatant immunodepleted with anti-Ac-K was detected [15, 27]. The primers for GDF15 promoter fragments were as follows: primer for −234 to −76 nt, forward: 5′-GGCTGGAATGGTGTCCT-3′, reverse: 5′-CCCCTGCCTGCAGAGTA-3′; primer for −103 to +58, forward: 5′-TCAAACAATCCACCCACCT-3′, reverse: 5′-TTTCCAGTCTAAGCAGGGTG-3′.

### His-pulldown assay

The His-pulldown assay was performed according to the manufacturer’s instruction of his-tag isolation & pulldown dynabeads. Briefly, the recombinant his-tagged GCN5 protein (5 μg) were added to 50 μl cobalt-coated magnetic beads and incubated on a roller at room temperature for 10 min. Then the bead/protein complex was washed and resuspended in the pulldown buffer (3.25 mM sodium-phosphate, 70 mM NaCl and 0.01% Tween-20). Subsequently, the flag-tagged KLF5 (5 μg) was added into the bead/protein complex incubated for 1 h. After washing, the mixture was boiled with loading buffer and analyzed by SDS-PAGE [48].

### In vitro acetylation assay

The active full-length recombinant GCN5 or heat-inactivate were incubated with the recombinant flag-KLF5 in 50 μl acetyltransferase assay buffer (50 mM Tris-HCl, pH 8.0, 10% glycerol, 0.1 mM EDTA, 1 mM PMSF, and 1 mM dithiothreitol) with 20 μM acetyl-CoA at 37 °C for 30 min. The acetylation of KLF5 was determined by IB using an acetylated lysine antibody [48, 49].

### Mass spectrometry analysis

A549 cells were overexpressed with flag-tagged KLF5 fusion protein and then subject to lysis reagent. Each clarified lysate was bound to anti-flag immunoaffinity resin, and then bounded protein complexes were competitively eluted using synthetic flag peptide. The purified flag-KLF5 protein was analyzed by mass spectrometer (ULTRAFLEX-II, Germany) at the Center of Hygienic Analysis & Detection of Nanjing Medical University [25, 50].

### Cell proliferation examination

For CCK8, the cells were allowed to grow in a 96-well plate. The optical density (OD) values were documented. For cell counting, the cells transfected with indicated plasmids were seeded at day 0 and digested at day 7, and the live cells were counted. For colony formation, cells were seeded in 6-well plate. On day 14, the cells were stained with 0.1 % crystal violet, and visible colonies were calculated.

### Xenograft tumor experiment

A total of 32 nude mice were randomly and blindly divided into four groups (*N* = 8 per group). The sample size was decided according to the estimation of the PASS software (www.ncss.com/software/pass/) and previous experience. A549 cells infected stably with LV-shRNAs or LV-shCTR were harvested. Then 5 × 10^6^ cells were inoculated subcutaneously into axilla of nude mice to generate an A549 lung adenocarcinoma-bearing mouse model. The tumor volumes were monitored every 4 days and calculated using following formula: volume = 0.5 × length × width^2^. Twenty eight days after inoculation, the mice were sacrificed, and the tumors were weighed. Additionally, KLF5, GCN5, GDF15 expression, and KLF5 acetylation in xenografts were detected by IP and IB, and the tumor cell proliferation was assessed by cyclin D1, PCNA, and Ki67 staining.

### Statistical analysis

Data are presented as means ± S.E.M. of three independent experiments. GraphPad Prism was used for data analysis. Two-tailed *t*-test was applied to determine significant differences between two groups while data among several groups were compared by one-way ANOVA test with Dunnett’s correction. The relationships between KLF5, GCN5, and GDF15 expression were analyzed by the Spearman’s correlation. The *χ*^2^ test was used for the correlations of KLF5, GCN5, GDF15, and C5aR expression in tumor tissues with clinic-pathological parameters of NSCLC patients. Statistics with *p* value < 0.05 were considered as statistically significant.

## Electronic supplementary material

Supplementary Data
