# Supplementary material for: C5a induces A549 cell proliferation of non-small cell lung cancer via GDF15 gene activation mediated by GCN5-dependent KLF5 acetylation
Source: Oncogene. 2018 May 18;37(35):4821–37. doi: 10.1038/s41388-018-0298-9 (PMC6117268; doi:10.1038/s41388-018-0298-9)
Supplement: Supplementary file 1 — Supplementary Data [file 41388_2018_298_MOESM1_ESM.doc]

**Supplementary figures and tables**

**
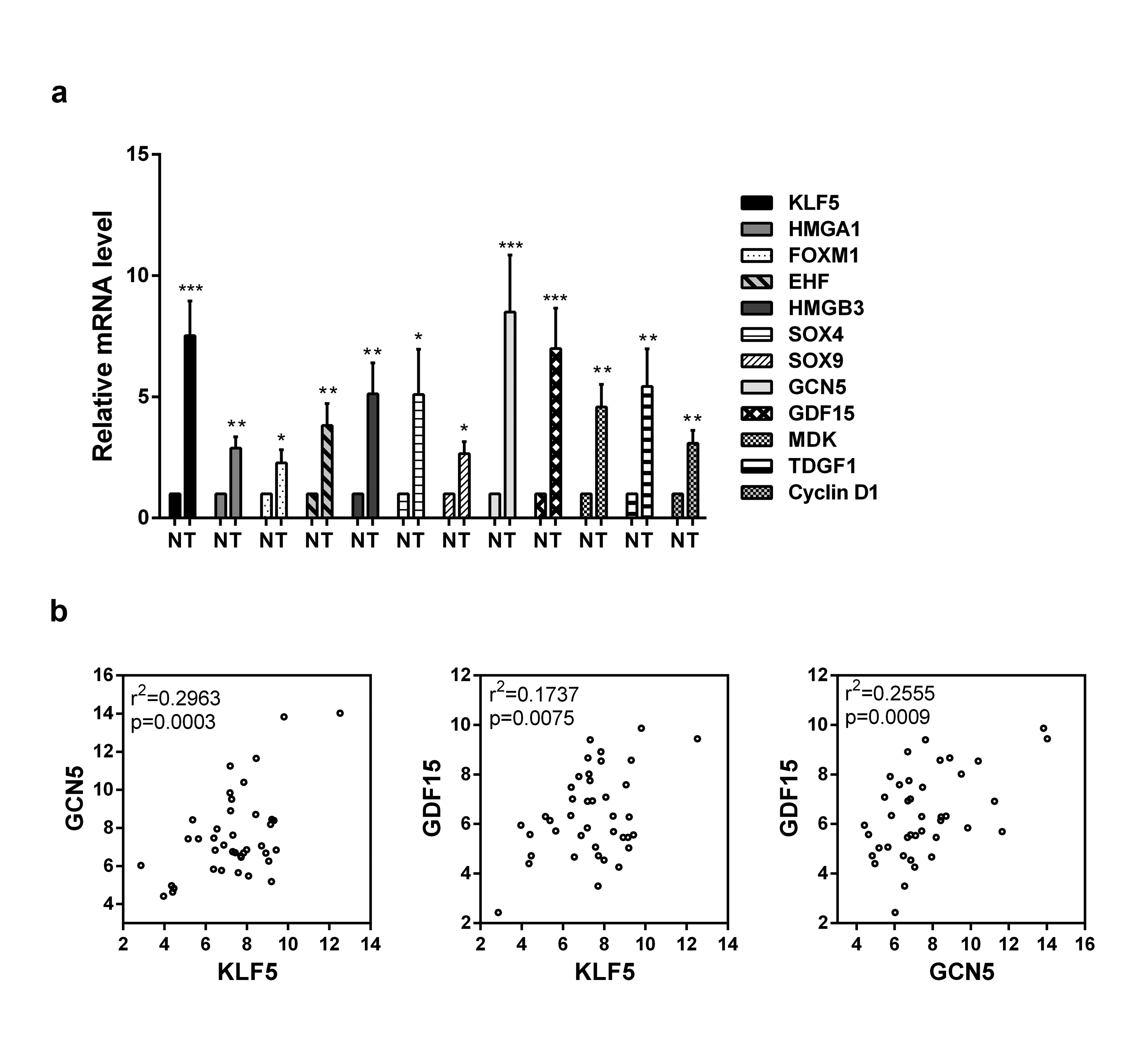
**

**Supplementary Figure 1.** **Quantification of proliferation-related genes by real-time PCR and the correlation between KLF5, GCN5 and GDF15.** **(a)** The mRNA change of the proliferation-related genes found by RNA-seq (i.e. KLF5, HMGA1, FOXM1, EHF, HMGB3, SOX4, SOX9, GCN5, GDF15, MDK, TDGF1 and cyclin D1) in 40 paired NSCLC fresh cancer tissues (N=40) was confirmed using real-time PCR. The mRNA levels of these genes were increased in NSCLC tissues, especially KLF5, GCN5 and GDF15 (**P*<0.05, ***P*<0.01, ****P*<0.001). **(b)** Correlation analysis showed a positive correlation between the mRNA levels of KLF5, GCN5 and GDF15.

**
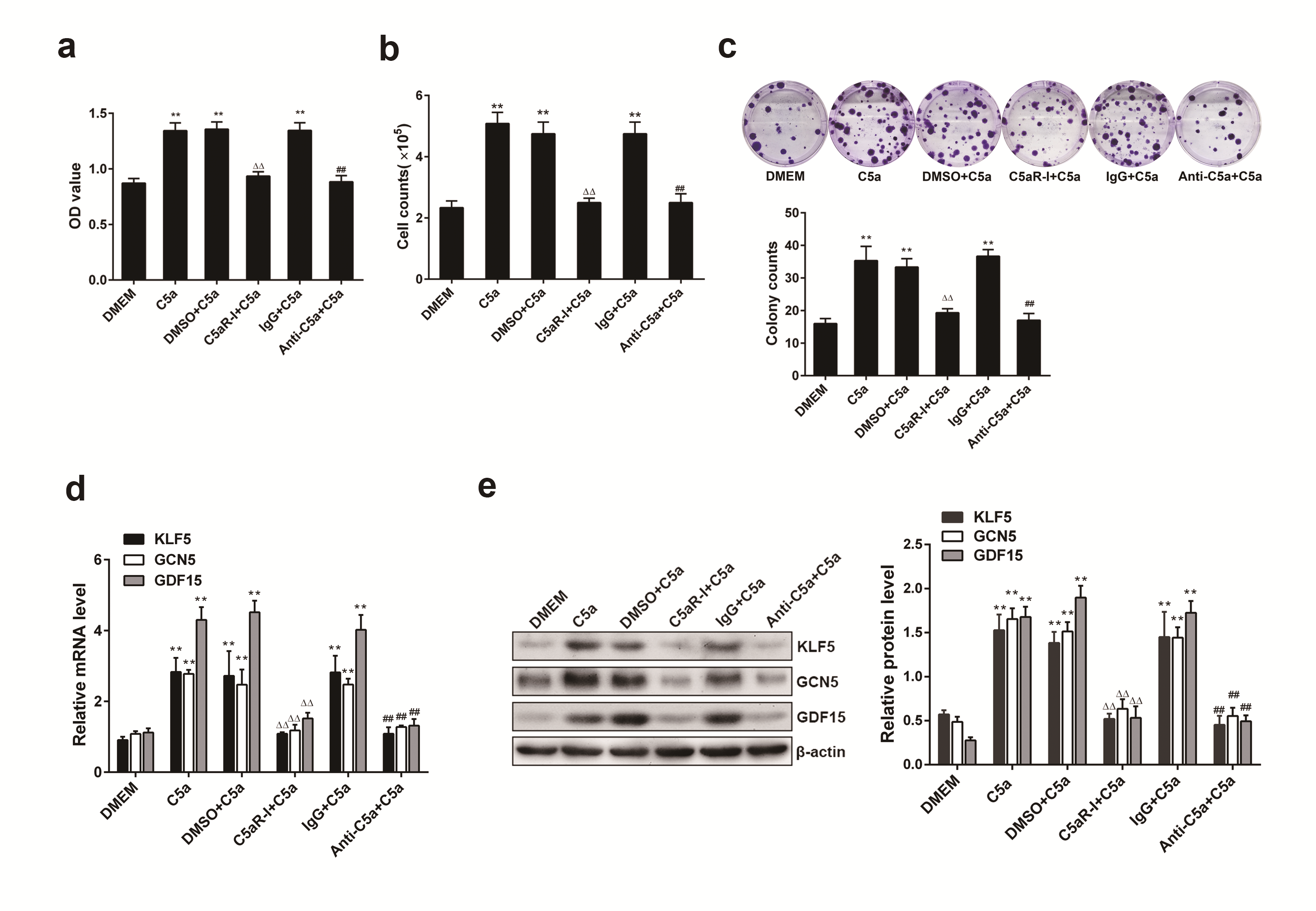
**

**Supplementary Figure 2. Roles of blocking C5aR by C5aR-inhibitor (C5aR-I) or neutralizing C5a by C5a antibody in C5a-inducing A549 cell proliferation and KLF5, GCN5 or GDF15 expression. (a-c)** A549 cells were pretreated with 1μM C5aR-I (W54011) or 40 ng/ml Anti-C5a for 30 min and then stimulated by C5a for 3h. CCK8 at 72h **(a)**, cell counting at 7d **(b)** and colony-forming assays at 14d **(c)** showed that the cell proliferation after C5a treatment were increased, but these increases were vanished in C5aR-I+C5a and Anti-C5a+C5a groups. **(d,e)** By real-time PCR **(d)** and IB **(e)**, the mRNA and protein of KLF5, GCN5 and GDF15 in A549 cells exposed to C5a for 3h were up-regulated, but down-regulated in C5aR-I+C5a and Anti-C5a+C5a groups. Representative photographs are shown. Data are represented as means ± S.E.M of three independent experiments. ***P*<0.01 vs. DMEM; △△*P*<0.01 vs. DMSO +C5a; ## *P*<0.01 vs. IgG+C5a.

**
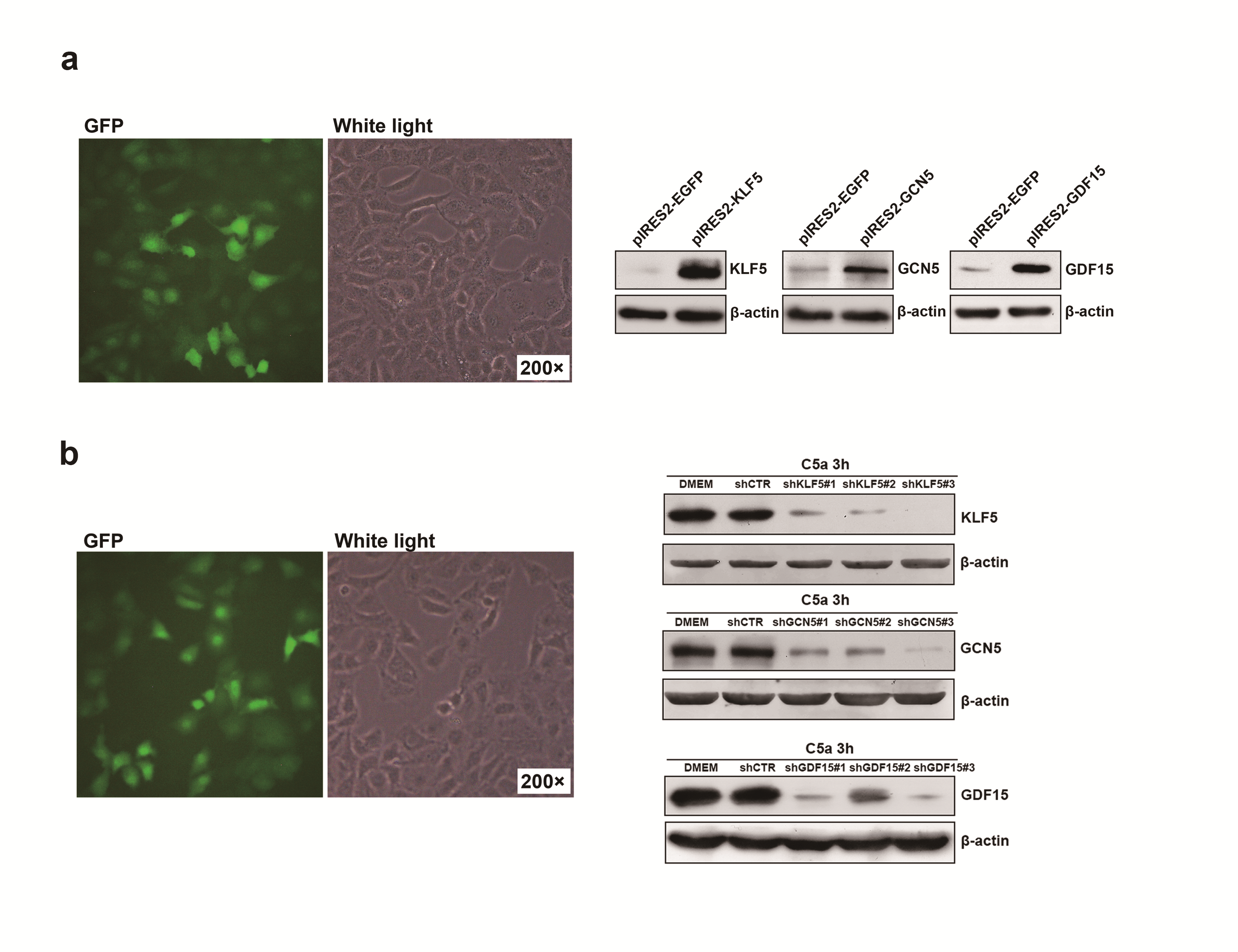
**

**Supplementary Figure 3. Expression of KLF5, GCN5, and GDF15 in A549 cells transfected with constructed plasmids. (a)** A549 cells were transfected with pIRES2-KLF5, pIRES2-GCN5 or pIRES2-GDF15 for 48h, and the levels of KLF5, GCN5 and GDF15 protein expression were examined. The transfection efficiency was determined by GFP expression at 24h in A549 cells (×200, left). IB displayed that A549 cells transfected with overexpression plasmids for 48h could significantly enhance the expression of KLF5, GCN5 and GDF15 proteins by contrast to pIRES2-EGFP group (right). **(b)** The cells were transfected with KLF5, GCN5 or GDF15 interference shRNA plasmids for 48h followed with C5a stimulation for 3h. Transfection efficiency was examined (left) and a significant reduction of these proteins were observed, particularly when No.3 (#3) plasmids of shKLF5, shGCN5 and shGDF15 were transfected (right). Representative pictures are exhibited. Results represent means ± S.E.M of three experiments.

**
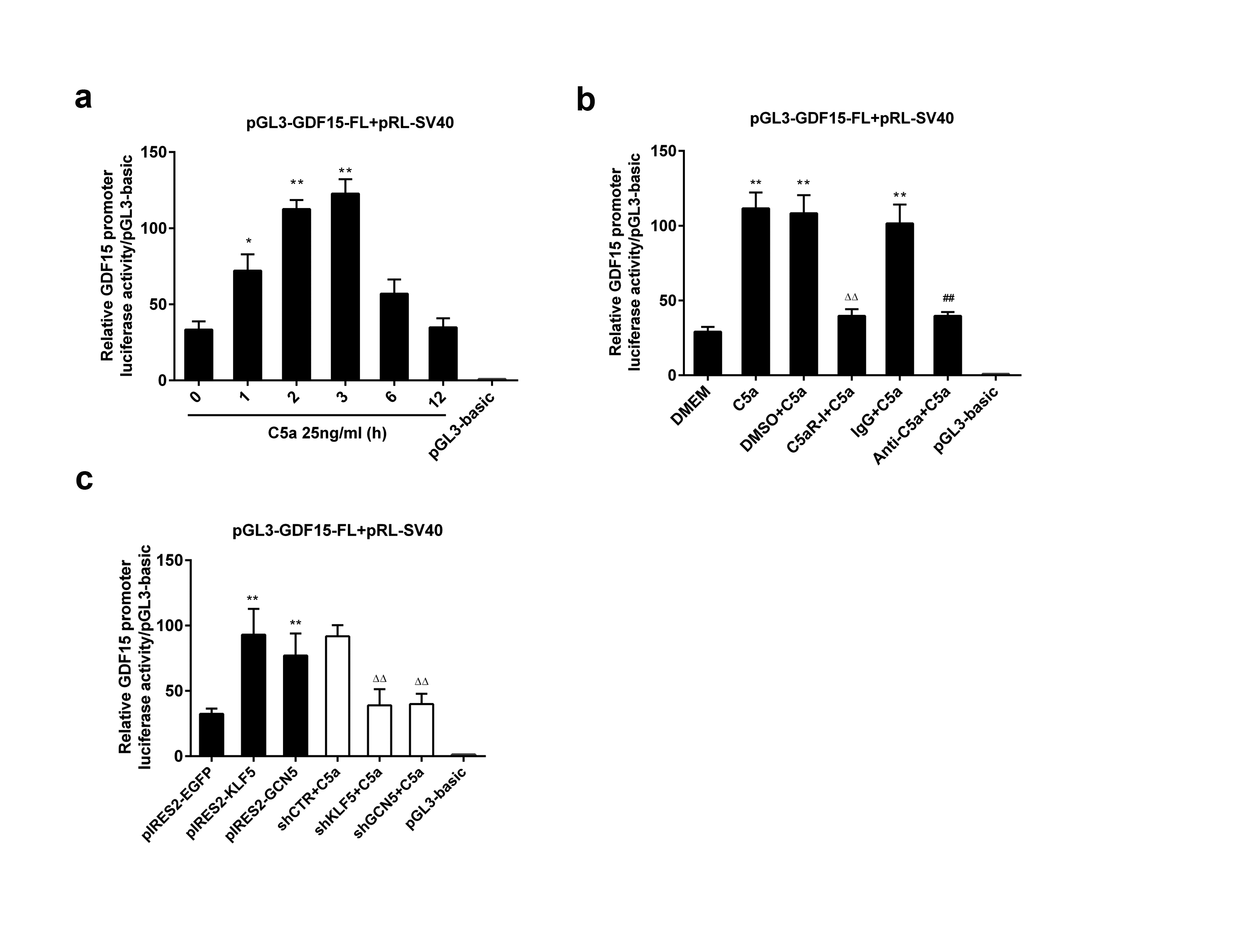
**

**Supplementary Figure 4．Change of GDF15 promoter activity in A549 cells with different treatment. (a)** Luciferase reporter analysis showed that GDF15 promoter activity in A549 cells upon C5a exposure was up-regulated at 1h, and peaked at 2h and 3h (**P*<0.05, ***P*<0.01 vs. 0h). **(b)** GDF15 promoter activity in A549 cells treated with C5a for 3h was markedly increased (***P*<0.01 vs. DMEM), and notably down-regulated by pretreatment of C5aR-I and Anti-C5a (△△*P*<0.01 vs. DMSO+C5a; ## *P*<0.01 vs. IgG+C5a). **(c)** A549 cells were co-transfected with corresponding plasmids and pGL3-GDF15-FL for 48h (followed with C5a for 3h when transfected with shRNA plasmids), and the promoter activity was examined by luciferase assay, and it showed a remarkable up-regulation in the cells transfected with pIRES2-KLF5 or pIRES2-GCN5 and a dramatic reduction when the cells were transfected with shKLF5 or shGCN5(***P*<0.01 vs. pIRES2-EGFP; △△*P*<0.01 vs. shCTR+C5a). Data expressed as means ± S.E.M from three independent experiments.

**
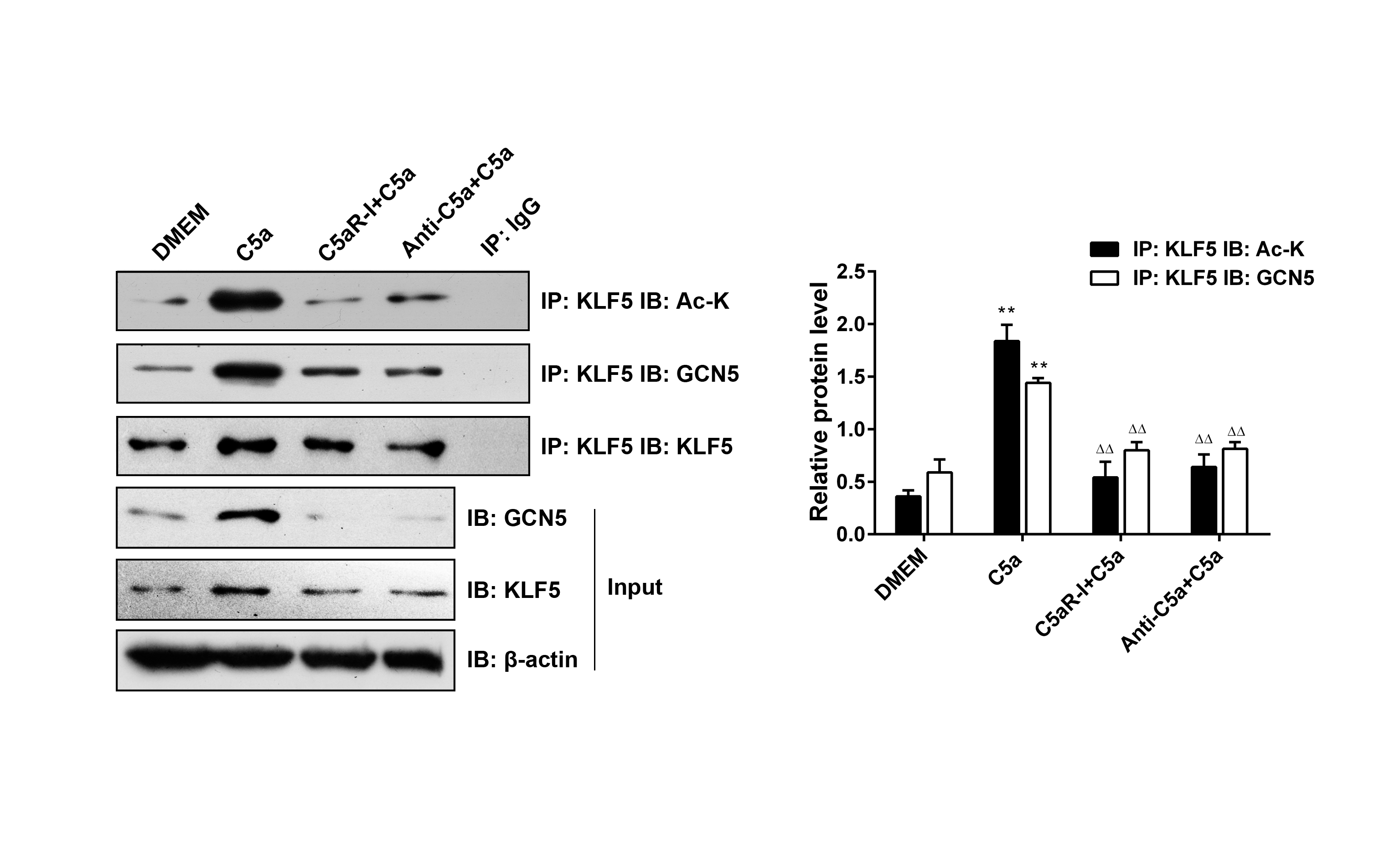
**

**Supplementary Figure 5. Role of blocking C5aR by C5aR-I or neutralizing C5a by C5a antibody in C5a-inducing KLF5 and GCN5 combination and KLF5 acetylation.** A549 cells treated with DMEM, C5a, C5aR-I+C5a and Anti-C5a+C5a, and the cell lysates were subjected to IP-IB using anti-KLF5 or GCN5 and Ac-K antibodies separately. The level of KLF5 binding to GCN5 and KLF5 acetylation in the cells upon C5a stimulation for 3h was elevated (***P*<0.01 vs. DMEM), but decreased in the cells treated with C5aR-I+C5a and Anti-C5a+C5a (△△*P*<0.01 vs. C5a). All data represent means ± S.E.M of three experiments. Representative photos are manifested.

**
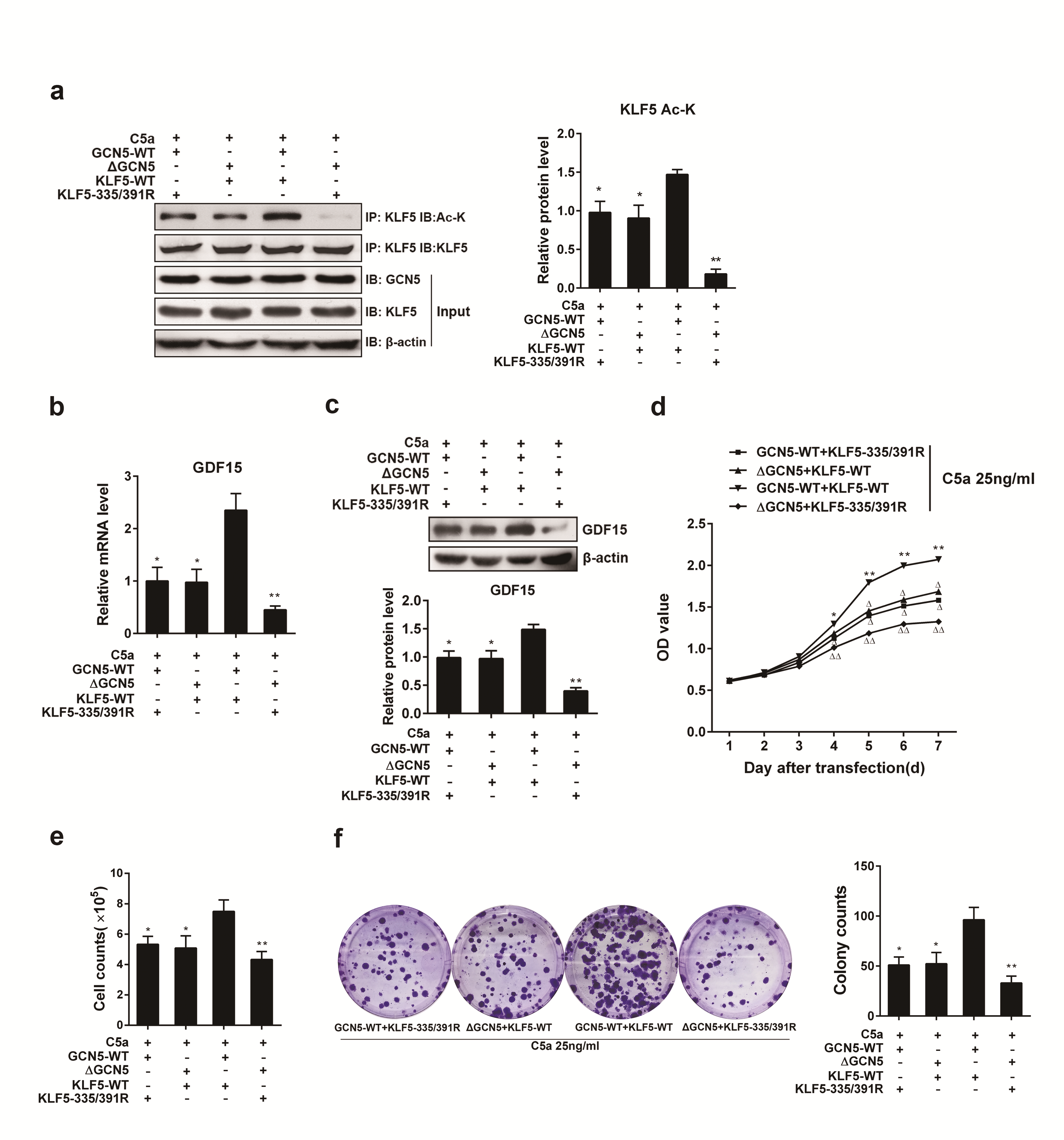
**

**Supplementary Figure 6. KLF5 acetylation, GDF15 expression and the proliferation of A549 cells co-transfected with the plasmids of wild-type (WT) or mutant of GCN5 and KLF5 upon C5a stimulation. (a)** A549 cells were co-transfected with wild-type or mutated KLF5 and GCN5 in different arrangements followed by C5a exposure for 3h. IB experiment showed that in comparison with GCN5-WT+KLF5-WT+C5a group, KLF5 acetylation was decreased in GCN5-WT+KLF5-K335/K391R+C5a, ΔGCN5 +KLF5-WT+C5a and ΔGCN5+KLF5-K335/K391R+C5a groups (**P*<0.05, ***P*<0.01). **(b,c)** Real-time PCR **(b)** and IB **(c)** assays manifested that change of GDF15 mRNA and protein was similar to KLF5 acetylation change (**P*<0.05, ***P*<0.01 vs. GCN5-WT+KLF5-WT+C5a). **(d-f)** The cell proliferation was detected by CCK8 **(d)**, cell counting **(e)** and colony formation **(f)**, and was shown down-regulated in GCN5-WT+KLF5-K335/K391R+C5a, ΔGCN5+KLF5-WT+C5a and ΔGCN5+KLF5-K335/K391R+C5a groups, especially more significantly in ΔGCN5+KLF5-K335/K391R+C5a group (**P*<0.05, ***P*<0.01 vs. GCN5-WT+KLF5-WT+C5a). Representative photographs are shown. All results are represented as means ± S.E. of three independent experiments.

**
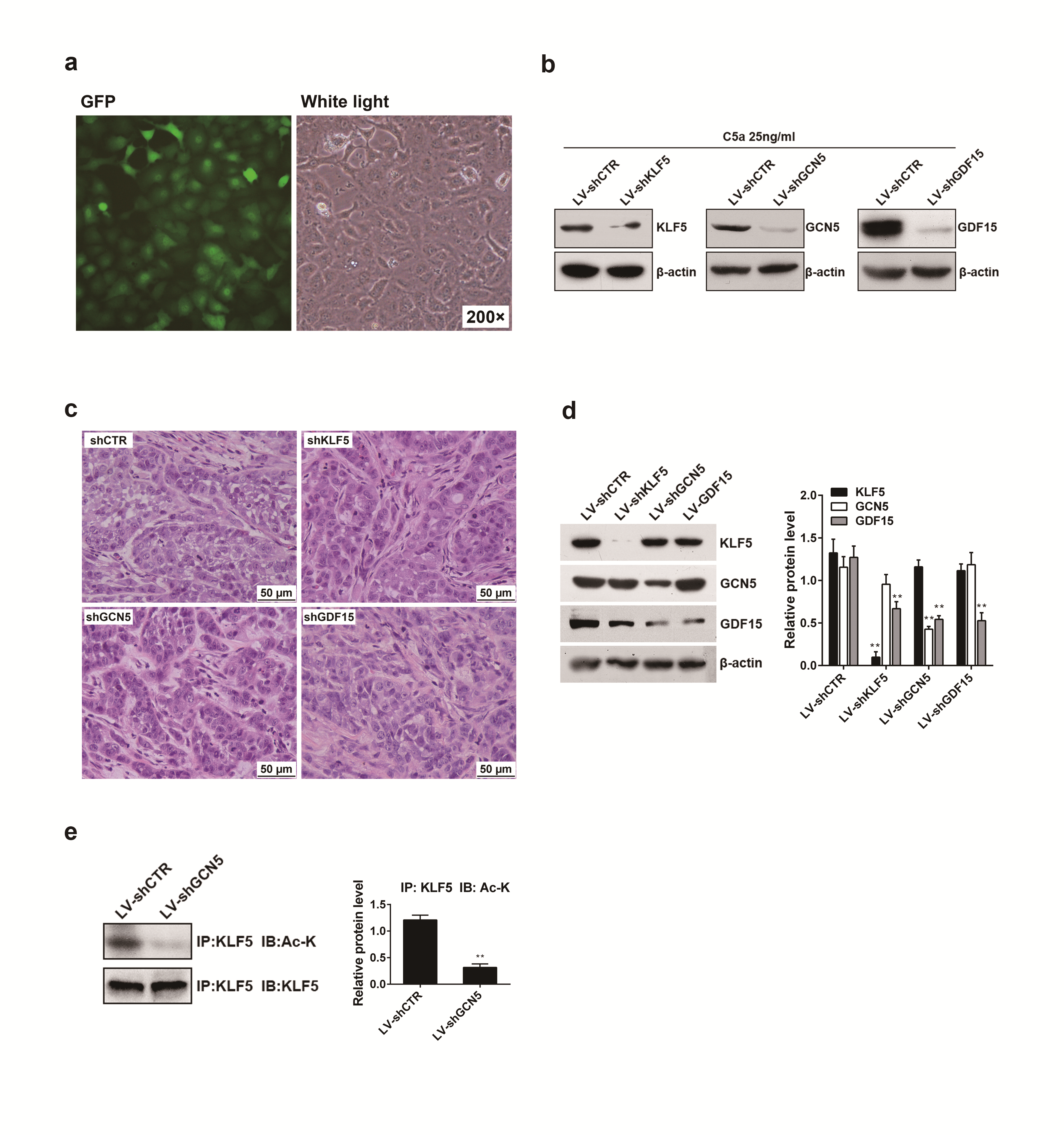
**

**Supplementary Figure 7. Effect of LV-shKLF5, LV-shGCN5 and LV-shGDF15 infection on corresponding protein expression, morphological change and KLF5, GCN5 or GDF15 expression including KLF5 acetylation in xenograft tumors. (a)** A549 cells (2×10^5^) was incubated with lentivirus (LV) at the titer of 4×10^6^ TU/ml for 48h and stable transfected cells were selected by puromycin. The infection efficiency was examined by GFP expression (×200). **(b)** IB analysis displayed that KLF5, GCN5 and GDF15 expression in A549 cells infected stably with corresponding LV-shRNAs and exposed to C5a for 3h was effectively down-regulated. **(c)** The tumors tissue sections of all mice with HE staining showed no significant differences on histomorphological changes. **(d)** IB enhibited that GDF15 protein was also attenuated in the xenograft tissues of the mice bearing LV-shKLF5, LV-shGCN5 and LV-shGDF15**.** **(e)** The KLF5 acetylation in xenograft tumors was detected by IP using anti-KLF5 and IB using an acetylated-lysine antibody. It displayed a significant reduction of KLF5 acetylation in xenograft of LV-shGCN5-bearing mice. Representative pictures are exhibited. Data are means ± S.E. of three independent experiments. ***P*<0.01 vs. LV-shCTR

**Supplementary Table 1** Primers for real-time PCR

| **Name** |  | **Primer (5’→3’)** |
| --- | --- | --- |
| β-actin | Forward | 5'-CAGCCATGTACGTTGCTATCCAGG-3' |
|  | Reverse | 5'-AGGTCCAGACGCAGGATGGCATG-3' |
| KLF5 | Forward | 5'-CCTGGTCCAGACAAGATGTG-3' |
|  | Reverse | 5'-GGGAGGAAGACGTTCATGTT-3' |
| HMGA1 | Forward | 5'-GCTGGTAGGGAGTCAGAAGG-3' |
|  | Reverse | 5'-TTGGTTTCCTTCCTGGAGTT-3' |
| FOXM1 | Forward | 5'-CAACCGCTACTTGACATTGG-3' |
|  | Reverse | 5'-CTGCTGTGATTCCAAGTGCT-3' |
| EHF | Forward | 5'-TGCAGCATCTGAAGTGGAAC-3' |
|  | Reverse | 5'-AGGAAGGTGACTGGTGGTTG-3' |
| HMGB3 | Forward | 5'-TACATCACTAAGGCGGCAAA-3' |
|  | Reverse | 5'-GCAACTTTAGCAGGACCCTT-3' |
| SOX4 | Forward | 5'-GCGACAAGATCCCTTTCATT-3' |
|  | Reverse | 5'-ACCGACCTTGTCTCCCTTC-3' |
| SOX9 | Forward | 5'-GTGCTCAAAGGCTACGACTG-3' |
|  | Reverse | 5'-AGAAGTCTCCAGAGCTTGCC-3' |
| GCN5 | Forward | 5'-CTGAAGACCATGACTGAGCGG-3' |
|  | Reverse | 5'-TCGGCCACAAAGAGCTTCC-3' |
| GDF15 | Forward | 5'-GCTACGAGGACCTGCTAACC-3' |
|  | Reverse | 5'-GCACTTCTGGCGTGAGTATC-3' |
| MDK | Forward | 5'-TACAATGCTCAGTGCCAGGA-3' |
|  | Reverse | 5'-CTTGGCGTCTAGTCCTTTCC-3' |
| TDGF1 | Forward | 5'-GCTAACGCCTCTTTTCCCCCTA-3' |
|  | Reverse | 5'-CCCGAGATGGACGAGCAAAT-3' |
| Cyclin D1 | Forward | 5'-GCCACTTGCATGTTCG-3' |
|  | Reverse | 5'-GGGCTCCTCAGGTTCA-3' |

**Supplementary Table 2** Primers for plasmid construction

| **Name** | **Primer( 5’→3’)** |  |
| --- | --- | --- |
| **KLF5** |  |  |
| Forward | 5'-CCGCTCGAGATGGCTACAAGGGTGCTGAG-3' |  |
| Reverse | 5'-CCGGAATTCTGGAACGGGTCACACGG-3' |  |
| **GCN5** |  |  |
| Forward | 5'-CCGCTCGAGATGGCGGAACCTTCCCAG-3' |  |
| Reverse | 5'-CCGGAATTCTCAGAATCCGAGGTGGAGACA-3' |  |
| **GDF15** |  |  |
| Forward | 5'-CCGCTCGAGATGCCCGGGCAAGAACT-3' |  |
| Reverse | 5'-CCGGAATTCTCATATGCAGTGGCAGTCTTTG-3' |  |
| **FLAG-KLF5** | |  |
| Forward | 5'-CCGCTCGAGATGGATTACAAGGATGACGACGATAAGGCTACAAGGG  TGCTGAGCATG-3' |  |
| Reverse | 5'-CCGGAATTCTTCTGGTGCCTCTTCATATGC-3' |  |
| **GDF15 promoter full length(-2068 to +103)** | |  |
| Forward | 5'-GGGGTACCGGAGTTTGGGGCCATATA-3' |  |
| Reverse | 5'-GAAGATCTATCCACGAGAGCACCAGCAA-3' |  |
| **GDF15 promoter truncate 1 (-1682 to +103)** | |  |
| Forward | 5'-GGGGTACCCATGTGCCACCACGACC-3' |  |
| **GDF15 promoter truncate 2 (-1039 to +103)** | |  |
| Forward | 5'-GGGGTACCTAATCCCACCACCAAGCC-3' |  |
| **GDF15 promoter truncate 3 (-401 to +103)** | |  |
| Forward | 5'-GGGGTACCTAGCCCGTTCTCCTCTGC-3' |  |
| **GDF15 promoter truncate 4 (-55 to +103)** | |  |
| Forward | 5'-GGGGTACCGGCGGAGACGGACAAAG-3' |  |

**Supplementary Table 3 The correlation between KLF5 expression and clinic-pathological** features in 185 cases of NSCLC patients

| Characteristics | Total | KLF5 expression | | p value^#^ |
| --- | --- | --- | --- | --- |
|  |  | Weak and negative | Strong |  |
|  | 185 | 65 | 120 |  |
| Sex |  |  |  |  |
| Male | 128 | 43(66.15) | 85(70.83) | 0.5103 |
| Female | 57 | 22(31.85) | 35(29.17) | |
|  |  |  |  |  |
| Age (years) | |  |  |  |
| <60 | 77 | 26(40.00) | 51(42.50) | 0.7574 |
| ≥60 | 108 | 39(60.00) | 69(57.50) | |
|  |  |  |  |  |
| Tumor size | |  |  |  |
| <5 cm | 117 | 58(89.23) | 59(24.17) | < 0.0001* |
| ≥5 cm | 68 | 7(10.77) | 61(50.83) | |
|  |  |  |  |  |
| Lymph node metastasis | | |  |  |
| Negative | 93 | 49(75.38) | 44(36.67) | < 0.0001* |
| Positive | 92 | 16(24.62) | 76(63.33) | |
|  |  |  |  |  |
| TNM stage | |  |  |  |
| I+II | 111 | 52(80) | 59(49.17) | < 0.0001* |
| III | 74 | 13(20) | 61(50.83) | |
|  |  |  |  |  |
| Pathologic type | |  |  |  |
| Squamous carcinoma | 70 | 24(36.92) | 46(38.33) | 0.1492 |
| Adenocarcinoma | 87 | 35(53.85) | 52(43.33) | |
| Large cell carcinoma | 13 | 1(1.54) | 12(10.00) | |
| Bronchioloalveolar carcinoma | 15 | 5(7.69) | 10(8.33) |  |

^#^chi-square test

*p<0.05

**Supplementary Table 4 The correlation between GCN5 expression and clinic-pathological** features in 185 cases of NSCLC patients

| Characteristics | Total | GCN5 expression | | p value^#^ |
| --- | --- | --- | --- | --- |
|  |  | Weak and negative | Strong |  |
|  | 185 | 76 | 109 |  |
| Sex |  |  |  |  |
| Male | 128 | 54(71.05) | 74(67.89) | 0.6467 |
| Female | 57 | 22(28.95) | 35(32.11) | |
|  |  |  |  |  |
| Age (years) |  |  |  |  |
| <60 | 77 | 30(39.47) | 47(43.12) | 0.6207 |
| ≥60 | 108 | 46(60.53) | 62(56.88) | |
|  |  |  |  |  |
| Tumor size |  |  |  |  |
| <5 cm | 117 | 56(73.68) | 61(55.96) | 0.0139* |
| ≥5 cm | 68 | 20(26.32) | 48(44.04) | |
|  |  |  |  |  |
| Lymph node metastasis |  |  |  |  |
| Negative | 93 | 46(60.53) | 47(43.12) | 0.0198* |
| Positive | 92 | 30(39.40) | 62(56.88) | |
|  |  |  |  |  |
| TNM stage |  |  |  |  |
| I+II | 111 | 53(69.74) | 58(53.21) | 0.0240* |
| III | 74 | 23(30.26) | 51(46.79) | |
|  |  |  |  |  |
| Pathologic type |  |  |  |  |
| Squamous carcinoma | 70 | 30(39.47) | 40(36.70) | 0.8298 |
| Adenocarcinoma | 87 | 35(46.05) | 52(47.71) | |
| Large cell carcinoma | 13 | 4(5.26) | 9(8.26) |  |
| Bronchioloalveolar carcinoma | 15 | 7(9.21) | 8(7.34) |  |

^#^chi-square test

*p<0.05

**Supplementary Table 5 The correlation between GDF15 expression and clinic-pathological** features in 185 cases of NSCLC patients

| Characteristics | Total | GDF15 expression | | p value^#^ |
| --- | --- | --- | --- | --- |
|  |  | Weak and negative | Strong |  |
|  | 185 | 70 | 115 |  |
| Sex |  |  |  |  |
| Male | 128 | 50(71.43) | 78(67.83) | 0.6068 |
| Female | 57 | 20(28.57) | 37(32.17) |  |
|  |  |  |  |  |
| Age (years) |  |  |  |  |
| <60 | 77 | 31(44.29) | 46(40) | 0.5735 |
| ≥60 | 108 | 39(55.71) | 69(60) |  |
|  |  |  |  |  |
| Tumor size |  |  |  |  |
| <5 cm | 117 | 51(72.86) | 66(57.39) | 0.0343* |
| ≥5 cm | 68 | 19(27.14) | 49(42.61) |  |
|  |  |  |  |  |
| Lymph node metastasis | |  |  |  |
| Negative | 93 | 50(71.43) | 43(37.39) | < 0.0001* |
| Positive | 92 | 20(28.57) | 72(62.61) |  |
|  |  |  |  |  |
| TNM stage |  |  |  |  |
| I+II | 111 | 51(72.86) | 60(52.17) | 0.0054* |
| III | 74 | 19(27.14) | 55(47.83) |  |
|  |  |  |  |  |
| Pathologic type |  |  |  |  |
| Squamous carcinoma | 70 | 24(34.29) | 46(40) | 0.2424 |
| Adenocarcinoma | 87 | 39(55.71) | 48(41.74) |  |
| Large cell carcinoma | 13 | 3(4.29) | 10(8.70) |  |
| Bronchioloalveolar carcinoma | 15 | 4(5.71) | 11(9.57) |  |

^#^chi-square test

*p<0.05

**Supplementary Table 6 The correlation between C5aR expression and clinic-pathological features in 185 cases of NSCLC patients**

| Characteristics | Total | C5aR expression | | p value^#^ |
| --- | --- | --- | --- | --- |
|  |  | Weak and negative | Strong |  |
|  | 185 | 81 | 104 |  |
| Sex |  |  |  |  |
| Male | 128 | 59(72.84) | 69(66.35) | 0.3426 |
| Female | 57 | 22(27.16) | 35(33.65) | |
|  |  |  |  |  |
| Age (years) |  |  |  |  |
| <60 | 77 | 35(43.21) | 42(40.38) | 0.6989 |
| ≥60 | 108 | 46(56.79) | 62(59.62) | |
|  |  |  |  |  |
| Tumor size |  |  |  |  |
| <5 cm | 117 | 63(77.78) | 54(51.92) | 0.0003* |
| ≥5 cm | 68 | 18(22.22) | 50(48.08) | |
|  |  |  |  |  |
| Lymph node metastasis | |  |  |  |
| Negative | 93 | 52(64.20) | 41(39.42) | 0.0008* |
| Positive | 92 | 29(35.80) | 63(60.58) | |
|  |  |  |  |  |
| TNM stage |  |  |  |  |
| I+II | 111 | 56(69.14) | 55(52.88)) | 0.0252* |
| III | 74 | 25(30.86) | 49(47.12) | |
|  |  |  |  |  |
| Pathologic type |  |  |  |  |
| Squamous carcinoma | 70 | 30(37.04) | 40(38.46) | 0.3026 |
| Adenocarcinoma | 87 | 42(51.85) | 45(43.27 ) | |
| Large cell carcinoma | 13 | 3(3.70) | 10(9.62) |  |
| Bronchioloalveolar carcinoma | 15 | 6(7.41) | 9(8.65) |  |

^#^chi-square test

*p<0.05
